# Furan-Indole-Chromenone-Based Organic Photocatalyst for α-Arylation of Enol Acetate and Free Radical Polymerization Under LED Irradiation

**DOI:** 10.3390/molecules30020265

**Published:** 2025-01-11

**Authors:** Aurélien Galibert-Guijarro, Adel Noon, Joumana Toufaily, Tayssir Hamieh, Eric Besson, Stéphane Gastaldi, Jacques Lalevée, Laurence Feray

**Affiliations:** 1 Aix Marseille Univ, CNRS, ICR, 13013 Marseille, France; aurelien.galibert@univ-amu.fr (A.G.-G.); eric.besson@univ-amu.fr (E.B.); stephane.gastaldi@univ-amu.fr (S.G.); 2 Université de Haute-Alsace, CNRS, IS2M UMR 7361, F-68100 Mulhouse, France; adelnoon@gmail.com (A.N.); t.hamieh@maastrichtuniversity.nl (T.H.); 3 Université de Strasbourg, F-67000 Strasbourg, France; 4 Laboratory of Materials, Catalysis, Environment and Analytical Methods (MCEMA), Faculty of Sciences, Doctoral School of Sciences and Technology (EDST), Lebanese University, Beirut 6573-14, Lebanon; joumana.toufaily@ul.edu.lb; 5 Faculty of Science and Engineering, Maastricht University, P.O. Box 616, 6200 MD Maastricht, The Netherlands

**Keywords:** organic photocatalyst, free radical polymerization, alkene arylation, photoredox catalysis, redox potential, photoinduced electron transfer

## Abstract

In this study we report on the efficiency of a furane-indole-chromenone-based organic derivative (**FIC**) as a photocatalyst in the α-arylation of enol acetate upon LED irradiation at 405 nm, and as a photoinitiator/photocatalyst in the free radical polymerization of an acrylate group in the presence of *bis*-(4-*tert*-butylphenyl)iodonium hexafluorophosphate (Iod) as an additive, or in the presence of both Iod and ethyl-4-(dimethyl amino) benzoate (EDB) under LED irradiation at 365 nm. The photochemical properties of this new light-sensitive compound are described, and the wide redox window (3.27 eV) and the high excited-state potentials FIC*/**FIC^●−^** (+2.64 V vs. SCE) and **FIC^●+^**/FIC* (−2.41 V vs. SCE) offered by this photocatalyst are revealed. The chemical mechanisms that govern the radical chemistry are discussed by means of different techniques, including fluorescence-quenching experiments, UV-visible absorption and fluorescence spectroscopy, and cyclic voltammetry analysis.

## 1. Introduction

**Scheme 1:**
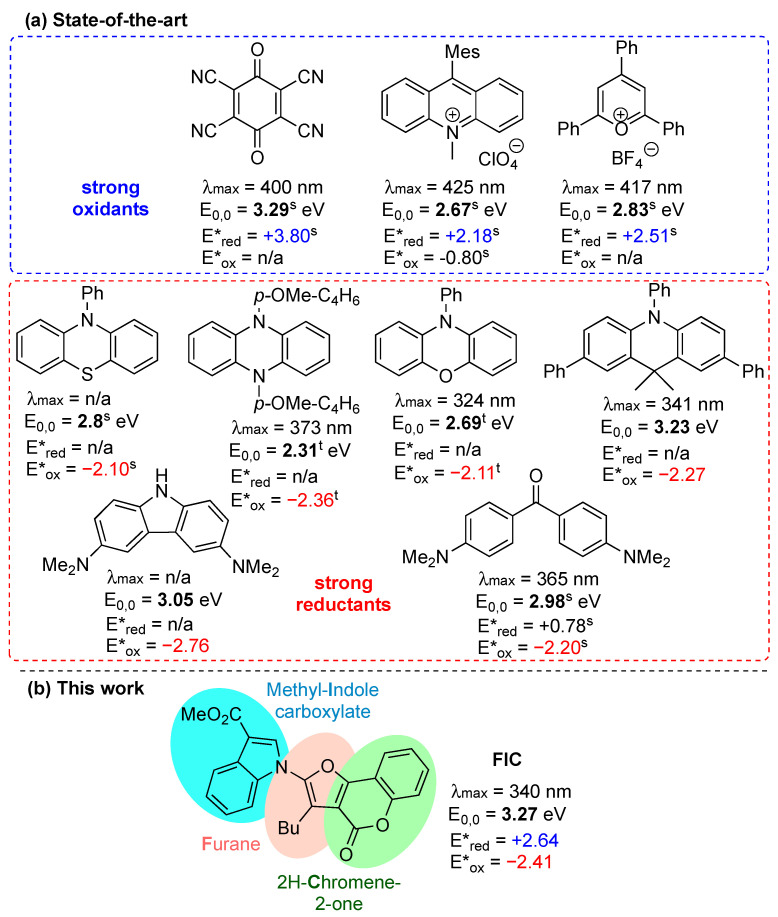
Organic photocatalysts (PCs) with high excited-state reduction and/or oxidation potentials E*_red_ (PC*/PC^●−^) > +2 V and/or E*_ox_ (PC^●+^/PC*) < −2 V vs. SCE. n/a: Data not available. ^s^: data for singlet excited state. ^t^: data for triplet excited state. Reported excited-state redox potentials E* V vs. SCE, see ref. [6].

Over the past decade, photochemical transformations have received increasing attention. In particular, photocatalyzed reactions using visible light as a photon source are now recognized as an essential and useful organic synthesis platform to access a wide range of complex molecular structures. In most visible-light photocatalyzed reactions, the activation mode involves a photocatalyst in its excited state being able to donate or take an electron from a substrate to form radical species [1,2]. Until recently, ruthenium- and iridium-based photocatalysts were the most representative in this field, but the need to find catalysts free of rare or toxic metals led organic chemists to take a closer look at photosensitive organic molecules, which present advantages in terms of cost and availability [3,4]. However, in relation to this field of research, there are still opportunities for further exploration, such as the development of more robust chromophores with highly reducing and/or oxidizing potentials (Scheme 1a) [5,6]. Thus, tuning the properties of already known architectures to increase their photocatalytic efficiencies, as well as to discover new photosensitive molecules, has become a challenging field of research [7,8].

Photosensitive organic molecules can also find applications in polymer chemistry [9,10]. Indeed, photopolymerization technologies have prevailed recently, increasing focus over other conventional industrial processes that offer low energy consumption and excellent time and spatial control without the use of volatile organic compounds [11,12]. This allows them to be widely implemented in many fields, including adhesives, coatings, 3D printing, dental materials, microelectronics, etc. [13,14,15]. The latest advancements in this context include the development of photoinitiating systems that employ visible light-emitting diodes (LEDs) as alternative irradiation sources to the traditional UV mercury lamps. The advantages offered by the former are low power consumption, low heat generation, low operating costs, longer emission wavelengths (and therefore more in-dept curing), safety, simplicity, portability, and easy availability [16,17]. Enormous effort has been dedicated to the development of new, highly efficient organic light sensitive molecules for applications both in organic synthesis and photopolymerizations.

**Scheme 2:**
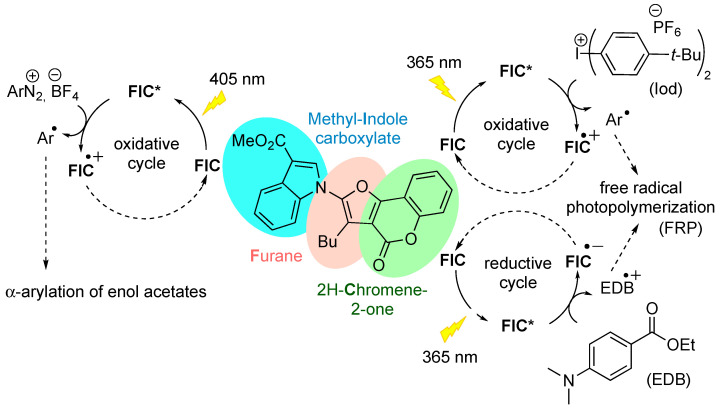
α-Arylation of enol acetate and free radical polymerization of acrylate group mediated by **FIC**.

During the course of our investigation on the reactivity of ynamides towards radical transformations, we have synthesized a persubstituted furane bearing methyl-indole carboxylate and 2H-chromene-2-one units [18], which we called **FIC** (Scheme 1b). As this compound contained aromatic rings and various groups or heteroatoms with acceptor and donor effects, we decided to investigate its behavior in photocatalyzed reactions, in particular its ability to promote the formation of aryl radicals from aryldiazonium and aryliodonium salts using an oxidative cycle. Thus, in this study, we report on the efficiency of **FIC** as a photocatalyst in the α-arylation of enol acetate under LED irradiation at 405 nm, and as a photoinitiating system in the free radical polymerization of an acrylate derivative in the presence of *bis*-(4-*tert*-butylphenyl)iodonium hexafluorophosphate (Iod) as an additive or in the presence of both Iod and ethyl-4-(dimethyl amino) benzoate (EDB) under LED irradiation (@365 nm) (Scheme 2). The chemical mechanisms governing the interaction between **FIC** with both additives (Iod and EDB) to produce initiating radicals have been fully detailed by performing fluorescence quenching experiments, combining UV-visible absorption and fluorescence spectroscopy, and cyclic voltammetry analysis.

## 2. Results and Discussion

**Scheme 3:**
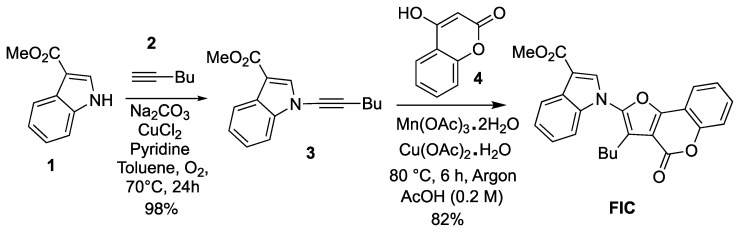
Two-step synthesis of **FIC**.

The synthesis of **FIC** can be achieved in two steps involving (i) cross-coupling reaction between commercially available 3-methyl indole carboxylate **1** and hex-1-yne **2** to give ynamide **3**, followed by (ii) Mn(III)/Cu(II)-mediated radical reaction of the latter with 4-hydroxycoumarine **4** leading to the desired compound (**FIC**) in an overall yield of 80% (Scheme 3) [18].

### 2.1. α-Arylation of Enol Acetate

Aryl diazonium salts are versatile building blocks in organic chemistry that can be used as powerful aryl radical precursors under various conditions [19,20]. Light-mediated arylation of alkenes, alkynes, enones [21], or enol acetates [22] has been achieved with diazonium salts in the presence of several metal- or organic-based photocatalysts, and can be considered as a reference reaction for the evaluation of a new photocatalyst.

**Scheme 4:**
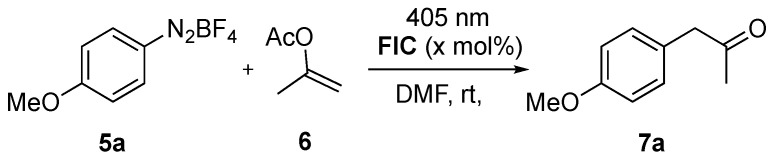
Arylation of enol acetate **6** with diazonium salt **5a**.

Arylation of isopropenyl acetate **6** with *p*-methoxybenzenediazonium tetrafluoroborate **5a** bearing a methoxy group was chosen as a model reaction to bring the first proof of concept (Scheme 4, Table 1). In the absence of **FIC**, after 2 h of irradiation at 405 nm in DMF, the solvent of choice to favor monoelectronic transfer [19], the reaction of diazonium salt **5a** with 10 equiv of enol acetate **6** led to a mixture of arylated expected ketone **7a**, and starting diazonium salt **5a** in a 20:80 ratio determined by ^1^H NMR carried out on the crude reaction mixture (Table 1, entry 1). The presence of 10 mol% of **FIC** clearly reversed the ratio in favor of **7a** (Table 1, entry 2). It should be noted that repeating the reaction in the dark led to no significant conversion, which emphasizes the importance of light in this process. Similar results were observed when the reaction was conducted in air or in the presence of one equivalent of TEMPO. Reactions involving **FIC** were also conducted in DMSO, leading to poor conversions of **5a** compared to those obtained in DMF. Optimized conditions, i.e., the use of 15 equiv of enol acetate in DMF, enabled the reaction to be completed in 4 h, leading to the formation of **7a** isolated in a yield of 80% after purification (Table 1, entry 4). Under the same conditions, without the photocatalyst, the diazonium salt was partially consumed and compound **7a** was isolated in a yield of 30% (Table 1, entry 3), showing the crucial role of **FIC** in this light-driven process.

**Scheme 5:**
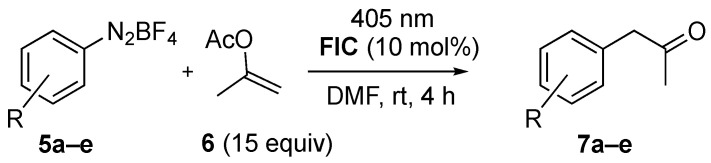
Arylation of enol acetate **6** with diazonium salts **5a–e**.

The scope of the transformation was studied by reacting enol acetate **6** with different diazonium tetrafluoroborate salts **5a–e** (Scheme 5, Table 2). Electron-donating as well as electron-withdrawing substituents were tested on the aromatic ring without loss of efficiency. The lowest yield was observed with ortho-substituted **5d**, probably due to steric hindrance (Table 2, entry 4).

**Scheme 6:**
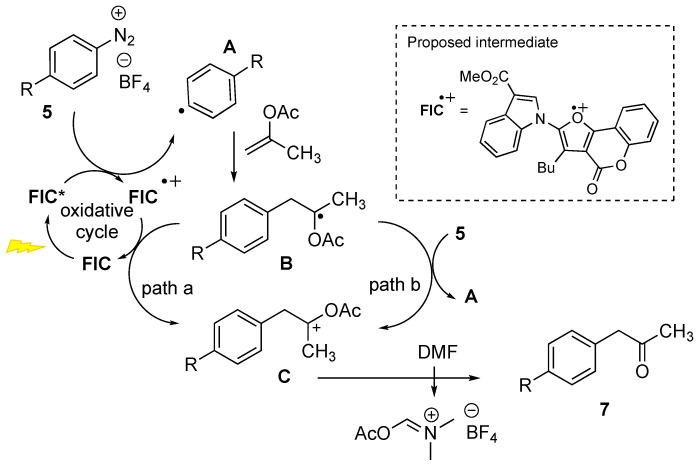
Mechanistic hypothesis for the formation of **7**.

The proposed mechanism for the entire process, based on our investigation and on previous reports [22], is depicted in Scheme 6. Upon light irradiation, the excited photocatalyst (FIC*) is oxidatively quenched by diazonium **5** to give an aryl radical intermediate **A** that can then be added regioselectively to the double bond of enol acetate **6**. At this stage, two pathways must be discussed. In path a, intermediate radical **B** can be oxidized by the photocatalyst (**FIC^●+^**) to regenerate **FIC** and give the intermediate carbocation **C**. According to previous literature [22], the latter can be deacetylated in the final step by DMF to generate the desired adduct **7**. In path a, **FIC** plays the role of a photocatalyst. In path b, radical intermediate **B** can react with starting diazonium **5** through a single electron transfer process to form intermediates **C** and **A**. In this last pathway, **FIC** plays the role of a photoinitiator only. It is important to note that in the absence of **FIC**, compound **7a** was still formed, but with a very low yield (Table 1, entry 3). This implies that the initiation step, i.e., the formation of the aryl radical **A**, may also be due to another mode of activation under irradiation [19]. However, the low yield obtained in this case argues in favor of path a in reactions where **FIC** is present, giving it the role of a real photocatalyst. Moreover, the fact that **FIC** can be recovered unchanged at the end of the reaction after purification on silica gel also supports this pathway.

### 2.2. Free Radical Polymerization (FRP) of Acrylates

Photopolymerization is now a highly efficient and versatile process that enables the rapid and sustainable fabrication of advanced materials, offering significant potential for applications in coatings, 3D printing, adhesives, and biomedical devices. This eco-friendly technique minimizes energy consumption and reduces waste, making it an ideal choice for greener industrial processes. Building on the high reactivity of **FIC** in organic synthesis, its potential application in photopolymerization processes has been thoroughly explored in mild LED irradiation conditions.

**Figure 1:**
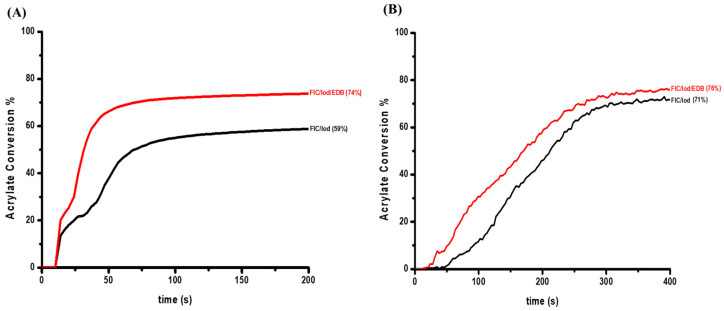

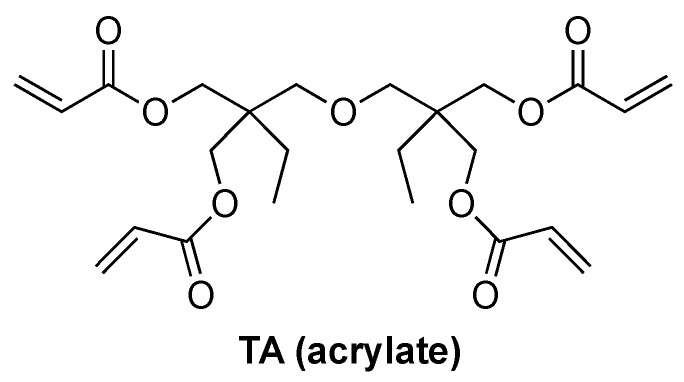
Photopolymerization profiles of **TA** (acrylate function conversion vs. irradiation time) upon exposure to a LED (λ = 365 nm) in the presence of **FIC**/Iod (0.5%/1% *w*/*w*) and **FIC**/Iod/EDB (0.5%/1%/1% *w*/*w*/*w*): (**A**) in laminate (thickness = 25 μm) and (**B**) in air (thickness = 2.3 mm). The irradiation starts at t = 10 s.

**Figure 2:**
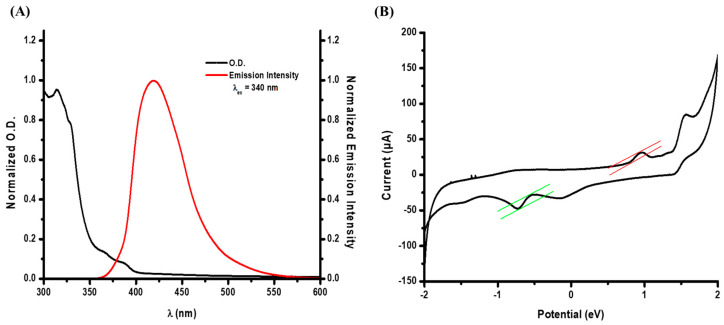
(**A**) UV-visible absorption and emission spectra of **FIC** in acetonitrile. (**B**) Cyclic voltammetry of **FIC** using tetrabutylammonium hexafluorophosphate dissolved in acetonitrile as the electrolyte.

The photoinitiation abilities and the associated final acrylate function conversions of the newly developed photocatalyst (**FIC**) in the presence of either Iod as an additive (0.5%/1% *w*/*w*) or Iod and EDB (0.5%/1%/1% *w*/*w*/*w*) for the FRP of di(trimethylolpropane)tetraacrylate (**TA**) monomer were tested in thin films (25 μm, in laminate; Figure 1A) and thick samples (2.3 mm, in air; Figure 1B), using irradiation with a 365 nm LED. These experiments were also conducted under irradiation at 405 nm, but the results were more satisfactory by using a 365 nm LED. which is attributed to the higher absorption properties of the **FIC** photocatalyst at λ = 365 nm (Figure 2A).

It is noteworthy to mention that when investigated separately, the **FIC** photocatalyst, Iod, or EDB could not initiate the FRP of TA upon exposure to a 365 nm LED (Figure S1, see Supporting Information) where low final conversions of the acrylate functional group were observed (28%, 33% and 28%, respectively). Moreover, even though the combination of Iod/EDB (without **FIC**) would lead to a Charge Transfer Complex (CTC), the detected polymerization rate obtained was slow (only 40% of acrylate functions conversion within 200 s of irradiation; Figure S1), clearly showing the importance of the combination of the **FIC** photocatalyst with the aforementioned additives for an efficient process.

**Scheme 7:**
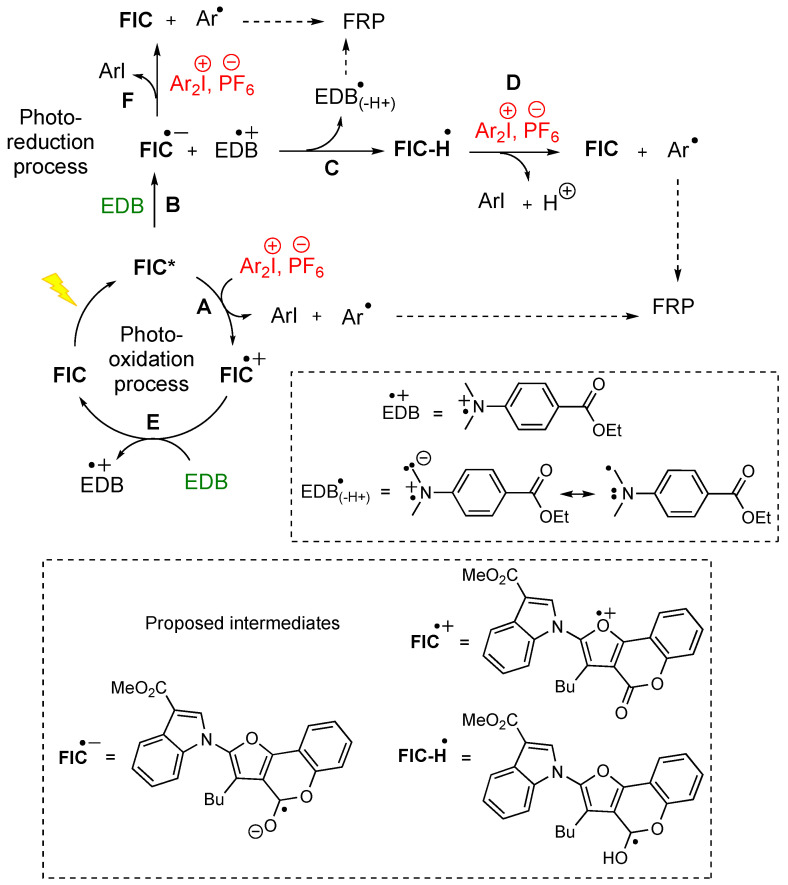
Mechanistic hypothesis for the FRP initiation.

A good photopolymerization profile is attained by combining **FIC** with Iod in both thin and thick samples, where a relatively high % of acrylate functions conversion were achieved (59% and 71% respectively). This improvement is related to the formation of aryl radicals (Ar^●^) by a photo-oxidation process where an electron is transferred from the excited state of the photocatalyst FIC* to Iod (step A, Scheme 7). These radicals are considered to be the initiating species for the FRP. Furthermore, the addition of a third component (EDB) to the previous combination (**FIC**/Iod) leads to an increase in the % of acrylate conversion to 74% in thin samples and 76% in thick samples. For a tetrafunctional monomer, the conversions achieved in acrylate functions are remarkably high. This marked enhancement is ascribed to the generation of EDB^●^_(–H+)_ radicals besides the formed aryl radicals, which are both responsible for initiating FRP. These EDB^●^_(–H+)_ radicals are formed by a photo-reduction process where an electron is transferred from EDB to the excited state of the photocatalyst FIC* (step B, Scheme 7) followed by a proton abstraction (step C, Scheme 7). Additionally, **FIC**^●+^ can react with EDB (step E, Scheme 7) or FIC-H^●^ with Iod (step D, Scheme 7) or FIC^●−^ with Iod (step F, Scheme 7), so that the photocatalyst **FIC** can be regenerated.

### 2.3. Photochemical Properties of FIC

To better understand the interaction between the newly investigated photocatalyst (**FIC**) and the additives (Iod and EDB), fluorescence quenching experiments, commonly used to show the involvement of bimolecular reactions from excited states [23], were carried out in acetonitrile. The concentration of Iod and EDB were increased cumulatively and each time a fluorescence spectrum was recorded.

**Figure 3:**
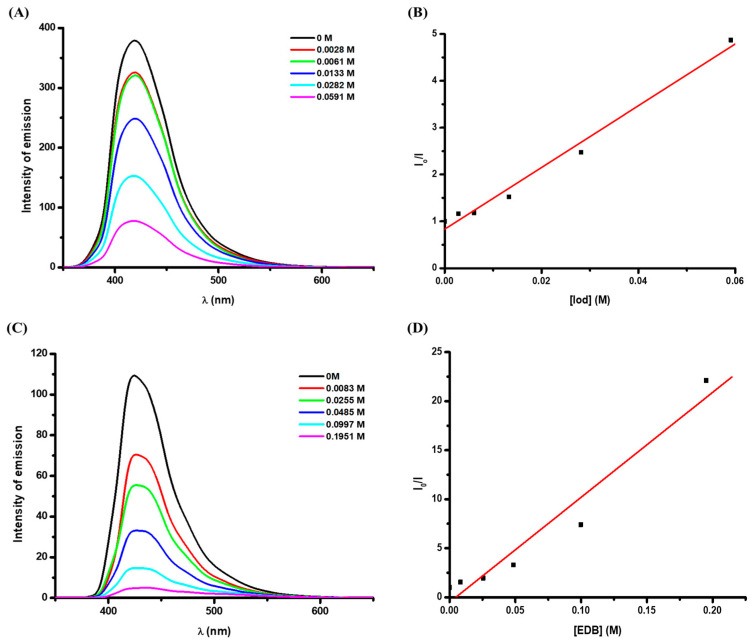
Fluorescence quenching study of **FIC**: (**A**) with Iod, and (**C**) with EDB in acetonitrile. Associated Stern–Volmer plot of **FIC**: (**B**) with Iod, and (**D**) with EDB.

Figure 3A,C show a prompt fluorescence quenching occurring for **FIC** with Iod and EDB, respectively, which evidently attest to the strong interaction of our photocatalyst (**FIC**) with both Iod and EDB (as shown in Scheme 7 (steps A and B)). Furthermore, the associated Stern–Volmer plots (Figure 3B,D) allow us to calculate the Stern–Volmer coefficients (K_sv_) and the electron transfer quantum yields (Φ_et_) [24], which are gathered in Table 3. High electron transfer quantum yields were obtained (0.8, and 0.95 for **FIC** with Iod and EDB, respectively) which also verifies the strong **FIC**/additives interaction and bimolecular electron transfer involving **FIC** and Iod, and **FIC** and EDB.

The singlet excited state energy E_S1_, determined at 3.27 eV from the crossing point of the absorption and fluorescence spectra (Figure 2A), and the oxidation and reduction potentials (E_ox_ and E_red_) determined by cyclic voltammetry (Figure 2B), allowed for the evaluation of the free energy change (ΔG) for the electron transfer reaction between **FIC** and the additives (Iod and EDB) (Table 3). Highly favorable ΔG are found in accordance with the strong **FIC**/Iod and strong **FIC**/EDB interactions observed in the fluorescence quenching experiments, supporting the proposed mechanism involving both the reduction of Iod and the oxidation of EDB (Scheme 7 (steps A and B)). Furthermore, the excited-state potentials FIC*/**FIC^●−^** (E*_red_ = +2.64 V vs. SCE) and **FIC^●+^**/FIC* (E*_ox_ = −2.41 V vs. SCE) calculated from these data using the very-well accepted method and equation in photochemistry [6,25] showed the powerful oxidative and reductive properties of **FIC** compared to already known metal- and organic-based photocatalysts (Scheme 1) [6,26]. To demonstrate these unique properties, other fluorescence bimolecular quenching experiments were conducted on **FIC** using substrates with high redox potentials (see Supplementary Materials and Table S1). Prompt fluorescence quenching occurred for **FIC** using acetophenone (E_red_ *=* −2.11 V vs. SCE) in acetonitrile, which attests to the strong interaction of our photocatalyst (**FIC**) with this substrate (see Figure S2) and bimolecular electron transfer involving **FIC** and acetophenone. The associated Stern–Volmer plots allowed us to calculate the Stern–Volmer coefficients (K_sv_) and the electron transfer quantum yields (Φ_et_ = 0.91). In addition, fluorescence quenching experiments conducted on **FIC** using anisole (E_ox_ *=* +1.75 V vs. SCE) and *o*-xylene (E_ox_ *=* +2.22 V vs. SCE) in cyclohexane (Figures S3 and S4) revealed the potential of our photocatalyst to also realize high-demanding oxidative processes. The high Stern–Volmer quenching verifies the favorable interaction between **FIC** and these substrates and bimolecular electron transfer involving **FIC** and anisole (Φ_et_ = 0.41), and **FIC** and *o*-xylene (Φ_et_ = 0.24).

## 3. Materials and Methods

**Synthesis of the studied Furane-Indole-Chromenone-based organic photocatalyst.** The synthesis of methyl 1-(3-butyl-4-oxo-4H-furo[3,2-c]chromen-2-yl)-1H-indole-3-carboxylate (**FIC**) was achieved in a two-step procedure using commercially available chemical compounds. Methyl indole-3-carboxylate (99%), 1-hexyne (97%) and 4-hydroxycoumarin were purchased from Sigma-Aldrich (St. Louis, MO, USA). The procedures are described in the Supplementary Materials.

**Other chemical compounds.** The chemical compounds used for the preparation of the resin mixtures were selected from the highest purity available and were used as received, and their chemical structures are represented. *Bis*-(4-*tert*-butylphenyl)iodonium hexafluorophosphate (Iod or SpeedCure 938) and ethyl-4-(dimethyl amino) benzoate were (Speedcure EDB) obtained from Lambson Ltd. (Wetherby, UK). The monomer used di(trimethylolpropane)tetraacrylate (TA) was obtained from Sartomer Europe (Paris, France). The storage stabilizer of the acrylate was not removed.

**Photopolymerization kinetics (RT-FTIR).** Detailed experimental conditions for each formulation have been cited in the captions of Figure 1 and Figure S1. The prepared formulations were stirred in the dark for 24 h. All polymerization experiments were performed using a 365 nm LED (incident light intensity at the sample surface I_0_ = 15 mW·cm^−2^) at room temperature, and the irradiation was initiated after t = 10 s. The weight of the system was calculated from the monomer content. The conversion of the acrylate function of TA was continuously followed by real-time Fourier transform infrared spectroscopy (JASCO FTIR 6600, Paris, France). For the thin samples (~25 μm in thickness), free radical polymerizations of TA were performed in laminate (the formulation is sandwiched between two polypropylene films to reduce the O_2_ inhibition). The decrease of C=C double bond band was continuously followed from 1581 to 1662 cm^−1^. For the thicker samples (~2.3 mm of thickness), the formulations were deposited on a polypropylene film inside a 2.3 mm mold in air. The evolution of the C=C band was continuously followed from 6117 to 6221 cm^−1^. The final acrylate function conversion of TA was obtained using the following equation:

$$FC\left(\%\right)=\frac{Ao-At}{Ao}\times 100$$

where FC is the final function conversion, A_0_ is the proportion of the peak area at 0 s, and A_t_ is the portion of the peak area at t s. The procedure has been already described by our group in reference [24].

**Steady state fluorescence.** The investigation of the fluorescence properties of **FIC** in acetonitrile was done using a JASCO FP-6200 spectrofluorimeter (Paris, France). Fluorescence quenching experiments on **FIC** using the cumulative addition of Iod or EDB (concentrations mentioned in Figure 2) were done by means of the JASCO FP-6200 spectrofluorimeter. The concentration of **FIC** used throughout the experiments was 5 × 10^−5^ M. The calculation of the Stern–Volmer coefficients (K_sv_) and the electron transfer quantum yields (Φ_et_) was done according to the equations mentioned in Table 3.

**UV-Visible absorption experiment.** The UV-visible absorption properties of **FIC** were examined for the calculation of the singlet excited state energy E_S1_ in acetonitrile using a JASCO V730 spectrometer (Paris, France). The concentration of **FIC** used throughout this experiment was 4 × 10^−5^ M.

**Oxidation and reduction potentials.** Oxidation and reduction potentials for **FIC** (E_ox_ and E_red_) were measured using cyclic voltammetry, with tetrabutylammonium hexafluorophosphate dissolved in acetonitrile as the electrolyte (potentials vs. Saturated Calomel Electrode—SCE). The free energy change (ΔG_et_) for the electron transfer reactions of **FIC** with both Iod and EDB were calculated using Equation (1), [12] where E_ox_, E_red_, E*, and C stand for the oxidation potential of the electron donor, the reduction potential of the electron acceptor, the considered excited state energy level and the coulombic term for the initially formed ion pair, respectively. Here, the reduction potential of Iod is E_red_(Iod) = −0.7 V, the oxidation potential of EDB is E_ox_(EDB) = 1 V, and C was neglected, as is usually done for polar solvents [12].

ΔG_et_ = E_ox_ − E_red_ − E* + C (1)

## 4. Conclusions

This study highlighted the photochemical properties of a member of a new family of light sensitive organic molecule. Furane-indole-chromenone-based organic derivative (**FIC**), which can be synthetized in a half-gram scale using a simple two-step procedure, has demonstrated its ability to behave as a photocatalyst in the α-arylation of enol acetate upon LED irradiation at 405 nm, and as a photoinitiator/photocatalyst in the free radical polymerization of an acrylate group in the presence of Iod as an additive or in the presence of both Iod and EDB under LED irradiation at 365 nm. The use of classical techniques such as fluorescence quenching experiments, UV-visible absorption and fluorescence spectroscopy, and cyclic voltammetry analysis enabled us to understand the mechanisms involved, and revealed the wide redox window (3.27 eV) and the high excited-state potentials (from −2.41 to +2.64 V) offered by this new photocatalyst, which can be engaged in both oxidative and reductive quenching processes. Future developments, related to the tuning of the properties of furane-indole-chromenone-based architecture that can be activated at longer wavelengths in photocatalyzed reactions, and free radical polymerization, will be proposed in upcoming studies. The use of more challenging substrates such as halogeno-arenes instead of diazonium or iodonium salts as a radical precursor will be reported soon.

## Figures and Tables

**Table 1:** Initial investigation: arylation of enol acetate **6** with diazonium salt **5a**.

Entry	6 (Equiv)	t (h)	FIC (mol%)	7a:5a ^1^	7a (%) ^2^
1	10	2	-	20:80	nd ^3^
2	10	2	10	70:30 ^4^	nd ^3^
3	15	4	-	-	30
4	15	4	10	-	80 ^5^

^1 1^H NMR ratio determined on the crude reaction mixture. ^2^ Isolated yield. ^3^ nd: not determined. ^4^ No conversion in ketone **7a** was observed when the reaction was conducted in the dark—in air—or in the presence of TEMPO (1 equiv). ^5^ A similar experiment was reported with Ru(II) as the photocatalyst to give a yield of 40% for **7a** (see ref. [22]).

**Table 2:** Scope of aryldiazonium tetrafluoroborate salts.

Entry	5	R	7 (%) ^1^
1	**5a**	4-OMe	**7a** (80)
2	**5b**	4-NO_2_	**7b** (77)
3	**5c**	H	**7c** (88)
4	**5d**	2-Br	**7d** (61)
5	**5e**	3-CN	**7e** (75)

^1^ Isolated yield.

**Table 3:** Parameters characterizing the chemical mechanisms associated with the **FIC**/Iod and **FIC**/EDB interactions in acetonitrile.

	E_S1_ (eV)	E_ox_ (V)	ΔG_S1(FIC/Iod)_ (eV) ^a^	E_red_ (V)	ΔG_S1(FIC/EDB)_ (eV) ^b^	K_sv (Iod)_ (M^−1^) ^c^	Φ_et (FIC/Iod)_ ^d^	K_sv (EDB)_ (M^−1^) ^c^	Φ_et (FIC/EDB)_ ^e^
FIC	3.27	0.86	−1.71	−0.63	−1.64	66	0.8	107	0.95

^a^ Evaluated from ∆G_S1 (PI/Iod)_ = E_ox_ − E_red(Iod)_ − E_S1_; E_red(Iod)_ = −0.7 eV. ^b^ Evaluated from ∆G_S1(PI/EDB)_ = E_ox(EDB)_ − E_red_ − E_S1_; E_ox (EDB)_ = 1 eV. ^c^ Stern–Volmer coefficient (K_sv_): slope of the quenching curve (I_0_/I = 1 + Ksv[Iod]). ^d^ Electron transfer quantum yield (Φ_et_): obtained according to the following equation: Φ_et_ = k_sv_[Iod]/(1 + k_sv_[Iod]) [24]. ^e^ Electron transfer quantum yield (Φ_et_): obtained according to the following equation: Φ_et_ = k_sv_[EDB]/(1 + k_sv_[EDB]) [24].

## Data Availability

The data underlying this study are available in the published article and its Supporting Information.

## Supplementary Materials

The following supporting information can be downloaded at: https://www.mdpi.com/article/10.3390/molecules30020265/s1, Figure S1: Photopolymerization profiles of TA; Figures S2, S3 and S4: Fluorescence quenchings for **FIC**. Table S1: parameters characterizing the **FIC**/electron donor or acceptor systems. Experimental procedures, product characterization data. References [27,28,29,30,31,32,33,34] are cited in the Supplementary Materials.
