# Supplementary material for: Furan-Indole-Chromenone-Based Organic Photocatalyst for α-Arylation of Enol Acetate and Free Radical Polymerization Under LED Irradiation
Source: Molecules. 2025 Jan 11;30(2):265. doi: 10.3390/molecules30020265 (PMC11767514; doi:10.3390/molecules30020265)
Supplement: Supplementary file 1 [file molecules-30-00265-s001.zip › molecules-3298625-supplementary.pdf]

Furan-indole-chromenone-based organic photocatalyst for α-arylation of enol acetate and free radical polymerization under LED irradiation

Aurélien Galibert-Guijarro [1], Adel Noon [2,3,4], Joumana Toufaily [4], Tayssir Hamieh [4,5], Eric Besson [1], Stéphane Gastaldi [1], Jacques Lalevée [2,3,*], Laurence Feray [1,*]

[1] Aix Marseille Univ, CNRS, ICR, Marseille, France.
[2] Université de Haute-Alsace, CNRS, IS2M UMR 7361, F-68100 Mulhouse, France.
[3] Université de Strasbourg, F-67000 Strasbourg, France.
[4] Laboratory of Materials, Catalysis, Environment and Analytical Methods (MCEMA), Faculty of Sciences, Doctoral School of Sciences and Technology (EDST), Lebanese University, Beirut 6573-14, Lebanon.
[5] Faculty of Science and Engineering, Maastricht University, P.O. Box 616, 6200 MD Maastricht, Netherlands.
* Correspondence: J.L. jacques.lalevee@uha.fr, L.F. laurence.feray@univ-amu.fr

Supplementary Materials

1 Experimental procedures S2
1.1 General S2
1.2 Synthesis of methyl 1-(3-butyl-4-oxo-4H-furo[3,2-c]chromen-2-yl)-1H-indole-3-carboxylate (**FIC**) S3
1.3 General Procedure for the preparation of aryl diazonium tetrafluoroborates **5**. S5
1.4 General procedure for the photocatalytic arylation of isopropenyl acetate. S8
2 Photopolymerization profiles of TA S11
3 Fluorescence quenching study of **FIC** (by acetophenone, anisole, and *o*-xylene) S12
4 ^{1}H and ^{13}C{^{1}H} NMR Spectra for 3, FIC, and 5e. ^{1}H for Spectra **5a-d**, **7a-e**. S14

1 Experimental procedures

1.1 General

Commercially available solvents and reagents were used as purchased. DMF was stored on molecular sieves. Analytical thin layer chromatography was performed on pre-coated silica gel plates. NMR spectra were recorded on a Bruker Avance III nanobay spectrometer with a BBFO+ probe at 300K at 400 (^{1}H) and 100 MHz (^{13}C) using $CDCl_3$ and DMSO-d_6 as solvents. Chemical shifts (δ) are reported in ppm. Signals due to residual protonated solvent or to the deuterated solvent served as the internal standards to calibrate the spectra. Multiplicity is indicated by one or more of the following descriptors: s (singlet), d (doublet), t (triplet), q (quartet), quint (quintet), m (multiplet), and br (broad). The *J* values are given in Hz.

High resolution MS mass spectrometry (MS) experiments were performed with a Waters Synapt G2 HDMS quadrupole/time-of-flight (Q/ToF) mass spectrometer (Manchester, UK), equipped with an electrospray ionization source operated in the positive ion mode using the following parameters: ESI capillary voltage: +2.8 kV; extraction cone voltage: +20 V; desolvation gas (N2) flow: 100 L h^{-1}; source temperature: 35°C; desolvation temperature: 35°C. In this hybrid instrument, ions were measured using an orthogonal acceleration time-of-flight (oa-TOF) mass analyzer. Internal mass calibration was systematically performed using two ions from a polymeric distribution. All peaks were centroided for mass measurement. Data analyses were conducted using the MassLynx 4.1 program provided by Waters.

1.2 Synthesis of methyl 1-(3-butyl-4-oxo-4H-furo[3,2-c]chromen-2-yl)-1H-indole-3-carboxylate (FIC)

Methyl 1-(hex-1-yn-1-yl)-1H-indole-3-carboxylate (3)

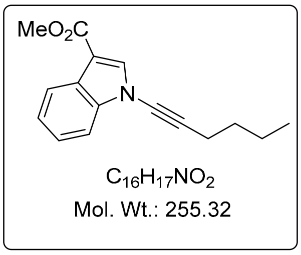

3

In a two-neck round-bottom flask equipped with a stir-bar, $CuCl_2$ (1.1 equiv, 4.4 mmol, 592 mg), methyl-indole-3-carboxylate **1** (5.0 equiv, 20 mmol, 3.50 g), Na_2CO_3 (2 equiv, 8 mmol, 848 mg) and dry toluene (20 mL) were combined. The reaction flask was purged with oxygen for 15 minutes. A solution of pyridine (2 equiv, 8 mmol, 0.64 mL) in dry toluene (20 mL) was added to the reaction flask via a syringe at room temperature. A balloon filled with oxygen was connected to the top of the refrigerant via a needle. The flask was placed in an oil bath and heated at 80°C. A solution of 1-hexyne **2** (1 equiv, 4 mmol, 0.45 mL) in dry toluene (20 mL) was added over 6h using a syringe pump. After the addition was completed, the reaction mixture was stirred at 70°C for 18h, and then cooled to room temperature. The crude mixture was concentrated in a vacuum and the residue was purified using flash chromatography on silica gel (pentane/ethyl acetate (97/03 to 80/20)), which led to ynamide **3** (990 mg, 3.9 mmol, 98 % yield) as a brown oil.

^{1}H NMR (400 MHz, $CDCl_3$) δ: 8.10-8.17 (pseudo d, *J* = 7.9, 1H), 7.88 (s, 1H), 7.50-7.54 (pseudo d, *J* = 8.2, 1H), 7.30-7.40 (m, 2H), 3.92 (s, 3H), 2.48 (t, *J* = 6.9, 2H), 1.59-1.65 (m, 2H), 1.48-1.53 (m, 2H), 0.98 (t, *J* = 7.3, 3H).

^{13}C{^{1}H} NMR (100 MHz, $CDCl_3$) δ: 164.4 (C=O), 138.5 (=C), 135.2 (=CH), 125.2 (=C), 124.2 (=CH), 123.3 (=CH), 121.8 (=CH), 111.4 (=CH), 109.9 (=C), 71.8 (≡C), 70.6 (≡C), 51.2 (CH_3), 30.8 (CH_2), 22.0 (CH_2), 18.0 (CH_2), 13.6 (CH_3).

HRMS (ESI): m/z: for [M+Na^+] $C_{16}H_{17}NO_2Na$ calcd: 278.1152, found: 278.1158.

Methyl 1-(3-butyl-4-oxo-4H-furo[3,2-c]chromen-2-yl)-1H-indole-3-carboxylate (FIC)

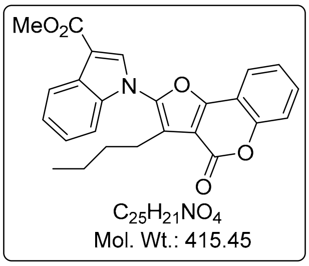

FIC

In a round-bottom flask equipped with a stir-bar and a refrigerant, methyl 1-(hex-1-yn-1-yl)-1H-indole-3-carboxylate **3** (1 equiv, 0.20 mmol, 50 mg), 4-hydroxycoumarin **4** (1.5 equiv, 0.24 mmol, 39 mg), $Mn(OAc)_3.2H_2O$ (3.3 equiv, 0.66 mmol, 177 mg), and $Cu(OAc)_2.H_2O$ (1 equiv, 0.2 mmol, 40 mg) were combined in an argon atmosphere. AcOH (0.2 M, 1 mL), previously degassed by bubbling argon for 30 minutes, was added to the reaction flask at room temperature. A balloon filled with argon was connected to the top of the refrigerant via a needle. The flask was placed in an oil bath and heated at 80 °C for 6 h and then cooled to room temperature and evaporated under reduced pressure. The residue was filtered on a neutral alumina solid phase with pentane (200 mL, Fraction 1) and ethyl acetate (600 mL, Fraction 2). The F_2 fraction was partially evaporated in a vacuum. Water was added and the layers were separated. An aqueous layer was extracted using AcOEt (3 x 15 ml). The combined organic phases were washed with water (20 mL), and dried over $MgSO_4$. The mixture was filtered and concentrated in a vacuum. The crude residue was purified using flash chromatography on a neutral alumina solid face, as needed. This procedure led to furan **FIC** (60 mg, 0.14 mmol, 73 % yield) as a yellow powder.

A scale up was performed with ynamide **3** (2 mmol, 500 mg), 4-hydroxycoumarin **4** (2.4 mmol, 390 mg), manganese triacetate (6.6 mmol, 1.78 g), and copper diacetate (2.0 mmol, 400 mg) in AcOH (10 mL) at 80°C for 6 h. This procedure led to furan **FIC** (681 mg, 1.64 mmol, 82% yield), which was isolated as a yellow powder.

^{1}H NMR (400 MHz, $CDCl_3$) δ: 8.27 (d, *J* = 7.3, 1H), 7.95 (s, 1H), 7.81 (dd, *J*=7.9 and 1.3, 1H), 7.53-7.58 (m, 1H), 7.46-7.50 (m, 1H), 7.33-7.42 (m, 3H), 7.27-7.30 (m, 1H), 3.98 (s, 3H), 2.69 (pseudo t, *J* = 7.6, 2H), 1.58 (quint, *J* = 7.4, 2H), 1.28 (sext, *J* = 7.5, 2H), 0.79 (t, *J* = 7.3, 3H).

^{13}C{^{1}H} NMR (100 MHz, $CDCl_3$) δ: 164.8 (C=O), 157.4 (=C), 155.4 (C=O), 153.1 (=C), 142.1 (=C), 138.1 (=C), 134.3 (=CH), 131.4 (=CH), 126.2 (=C), 124.7 (=CH), 124.6 (=CH),

123.4 (=CH), 122.2 (=CH), 121.1 (=CH), 119.2 (=C), 117.5 (=CH), 112.3 (=C), 111.5 (=C), 110.9 (=CH), 110.8 (=C), 51.4 (CH_3), 31.4 (CH_2), 22.8 (CH_2), 22.2 (CH_2), 13.6 (CH_3).

HRMS (ESI): m/z: for [$M+H^+$] $C_{25}H_{22}NO_5$ calcd: 416.1492; found: 416.1490.

1.3 General Procedure for the preparation of aryl diazonium tetrafluoroborates 5.

Aryl diazonium tetrafluoroborates were prepared according to the literature.[1] The appropriate aniline (1 equiv) was dissolved in a mixture of 1 mL of water and 1.9 mL of 50% hydrofluoroboric acid. After the reaction mixture was cooled to 0 °C using an ice bath, a solution of sodium nitrite (2 equiv) in water was added drop by drop. The suspension was stirred for an additional 40 min and then filtered, and the resulting solid was successively washed with a small amount of ice water, ethanol and diethyl ether. Finally, the product was dried in a vacuum for 10 minutes.

4-Methoxybenzenediazonium tetrafluoroborate (5a)[2]

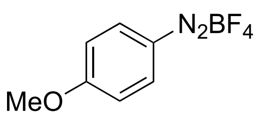

$C_7H_7BF_4N_2O$

Mol. Wt: 221.95

5a

The reaction was conducted according to the general procedure in the presence of *p*-anisidine (5 mmol, 616 mg), a mixture of 1 mL of water and 1.9 mL of 50% hydrofluoroboric acid, and Na_2NO_2 (680 mg in 1.5 mL). 4-Methoxybenzenediazonium tetrafluoroborate **5a** (380 mg, 1.7 mmol, 34% yield) was isolated as a grey powder.

^{1}H NMR (400 MHz; DMSO-d$_6$) δ: 8.61 (d, J = 9.4, 2H), 7.48 (d, J = 9.4, 2H), 4.04 (s, 3H).

[1] Roe, A. Preparation of aromatic fluorine compounds from diazonium fluoborates. The Schiemann reaction. *Org. React.* **1949**, *5*, 193.

4-Nitrobenzenediazonium tetrafluoroborate (5b)[2]

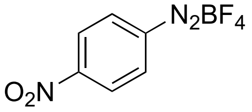

$C_6H_4BF_4N_3O_2$

Mol. Wt: 236.92

5b

The reaction was conducted according to the general procedure in the presence of nitraniline (5 mmol, 691 mg), a mixture of 1 mL of water and 1.9 mL of 50% hydrofluoroboric acid, and Na_2NO_2 (680 mg in 1.5 mL). 4-Nitrobenzenediazonium tetrafluoroborate **5b** (1.18g, 5 mmol, up to 99% yield) was isolated as a yellow powder.

^{1}H NMR (400 MHz; DMSO-d$_6$) δ: 8.93 (d, *J* = 9.4, 2H), 8.71 (d, *J* = 9.3, 2H).

Benzenediazonium tetrafluoroborate (5c)[2]

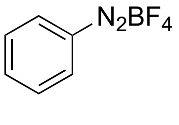

$C_6H_5BF_4N_2$

Mol. Wt: 191.92

5c

The reaction was conducted according to the general procedure in the presence of aniline (5 mmol, 466 mg), a mixture of 1 mL of water and 1.9 mL of 50% hydrofluoroboric acid, and Na_2NO_2 (680 mg in 1.5 mL). Benzenediazonium tetrafluoroborate **5c** (376 mg, 2.0 mmol, 39% yield) was isolated as a pink powder.

^{1}H NMR (400 MHz; DMSO-d$_6$) δ: 8.67 (d, *J* = 7.8, 2H), 8.21-8.31 (m, 1H), 7.92-8.04 (m, 2H).

[2] Tang, Z.Y.; Zhang, Y.; Wang, T.; Wang, W. Rhodium (I)-catalyzed synthesis of aryltriethoxysilanes from arenediazonium tosylate salts with triethoxysilane. *Synlett* **2010**, *5*, 804–808.

2-Bromobenzenediazonium tetrafluoroborate (5d)[3]

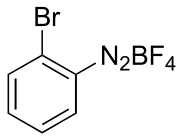

$C_6H_4BBrF_4N_2$

Mol. Wt: 270.82

5d

The reaction was conducted according to the general procedure in the presence of 2-bromoaniline (5 mmol, 861 mg), a mixture of 1 mL of water and 1.9 mL of 50% hydrofluoroboric acid, and Na_2NO_2 (680 mg in 1.5 mL). 2-Bromobenzenediazonium tetrafluoroborate **5d** (1.33g, 4.9 mmol, 98% yield) was isolated as a brown powder.

^{1}H NMR (400 MHz; DMSO-d$_6$) δ: 8.84 (dd, *J* = 8.3 and 1.5, 1H), 8.31 (dd, *J* = 8.2 and 0.8, 1H), 8.17 (td, *J* = 7.7 and 1.6, 1H), 7.99 (td, *J* = 8.4 and 1.0, 1H).

3-Cyanobenzenediazonium tetrafluoroborate (5e)

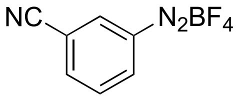

$C_6H_4BF_4N_3$

Mol. Wt: 216.93

5e

The reaction was conducted according to the general procedure in the presence of 3-cyanoaniline (5 mmol, 591 mg), a mixture of 1 mL of water and 1.9 mL of 50%

[3] Xu, Y.; Alcock, N.W.; Clarkson, G.J.; Docherty, G.; Woodward, G.; Wills, M. Woodward, G.; Wills, M. Asymmetric hydrogenation of ketones using a ruthenium (II) catalyst containing binol-derived monodonor phosphorus-donor ligands. *Org. Lett.* **2004**, *6*, 4105–4107.

hydrofluoroboric acid, and Na_2NO_2 (680 mg in 1.5 mL). 3-Cyanobenzenediazonium Tetrafluoroborate **6e** (651 mg, 3.0 mmol, 60% yield) was obtained as a brown powder.

^{1}H NMR (400 MHz; DMSO-d$_6$) δ: 9.22 (t, *J* = 1.6, 1H), 8.94 (dt, *J* = 8.5 and 1.1, 1H), 8.71 (dt, *J* = 8.0 and 1.1, 1H), 8.18 (t, *J* = 8.2, 1H).

^{13}C{^{1}H} NMR (100MHz; DMSO-d$_6$) δ: 143.9 (=CH), 136.6 (=CH), 136.1 (=CH), 132.4 (=CH), 118.3 (≡C), 115.5 (=C), 113.8 (=C).

HRMS (ESI): m/z: for [$2C^+ + A^-$] $C_{14}H_8BF_4N_6^+$ calcd: 347.0837, found: 347.0835.

1.4 General procedure for the photocatalytic arylation of isopropenyl acetate.

In a 5 mL snap vial, aryl diazonium tetrafluoroborate **5a-e (**1 equiv) and isopropenyl acetate **6** (15 equiv) were dissolved in 670 µL of dry DMF in the presence (condition a) or in the absence (condition b) of 0.01 equiv of the photocatalyst **FIC**. The vial was sealed with a septum and degassed via the "freeze-pump-method" (3×). The reaction mixture was stirred and irradiated for 2 to 4 h at a distance of ~10 cm (light intensity: 16.7 mW/cm^2 at 10 cm distance) with 18 W blue LEDs (Evoluchem® lamps, 405 nm) at 20°C. Then, the mixture was diluted with water and diethyl ether. The organic layer was washed three times with brine. Finally, the organic layer was dried over Na_2SO_4, filtered, and concentrated in a vacuum. Crude mixtures were purified by a column chromatograph packed with silica gel using petroleum ether/ethyl acetate (90:10 to 70:30) as an eluent to obtain the desired pure products **7**.

4-Methoxyphenylacetone (7a)[4]

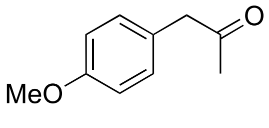

$C_{10}H_{12}O_2$

Mol. Wt: 164.20

7a

[4] Li, L.; Cai, P.; Guo, Q.; Xue, S.J. Et_2Zn-mediated rearrangement of bromohydrins. *Org. Chem.* **2008**, *73*, 3516–3522.

The reaction was conducted according to the general procedure in the presence of 4-methoxybenzenediazonium tetrafluoroborate **5a** (0.3 mmol, 67 mg), isoprenyl acetate **6** (4.5 mmol, 450 mg), and photocatalyst **FIC** (0.03 mmol, 11 mg) dissolved in 670 µL dry DMF. The mixture was irradiated for 4 h and the product was obtained after purification using flash chromatography on silica gel, leading to 4-methoxyphenylacetone **7a** (condition a: 40 mg, 0.24 mmol, 80% yield) (condition b: 15 mg, 0.09 mmol, 30%) as a colorless oil.

^{1}H NMR (400 MHz; DMSO-d$_6$) δ: 7.09 (d, *J* = 8.6, 2H), 6.87 (d, *J* = 8.7, 2H), 3.72 (s, 3H), 3.66 (s, 2H), 2.09 (s, 3H).

4-Nitrophenylacetone (7b)[5]

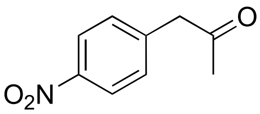

$C_9H_9NO_3$

Mol. Wt: 179.18

7b

The reaction was conducted according to the general procedure in the presence of 4-nitrobenzenediazonium tetrafluoroborate **5b** (0.3 mmol, 71 mg), isoprenyl acetate **6** (4.5 mmol, 450 mg), and photocatalyst **FIC** (0.03 mmol, 11 mg) dissolved in 670 µL dry DMF. The mixture was irradiated for 4 h and the product was obtained after purification using flash chromatography on silica gel, leading to 4-nitrophenylacetone **7b** (condition a: 37 mg, 0.21 mmol, 77% yield) as a pink powder.

^{1}H NMR (400 MHz; CDCl$_3$) δ: 8.17 (d, *J* = 8.7, 2H), 7.45 (d, *J* = 8.8, 2H), 3.84 (s, 2H), 2.19 (s, 3H).

Phenylacetone (7c)[6]

---

[5] Molinaro, C.; Mowat, J.; Gosselin, F.; O'Shea, P.D.; Marcoux, J.-F.; Angelaud, R.; Davies, I.W.J. A practical synthesis of α-aryl methyl ketones via a transition-metal-free meerwein arylation. *Org. Chem.* **2007**, *72*, 1856–1858.

[6] Yuan, L.Z.; Zhao, G.; Hamze, A.; Alami, M.; Provot, O. Chlorotrimethylsilane and Sodium Iodide: A Useful Combination for the Regioselective Deoxygenation of Arylalkyl-α-Diketones. *Adv. Synth. Catal.* **2017**, *359*, 2682–2691.

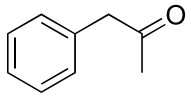

$C_9H_{10}O$

Mol. Wt: 134.18

7c

The reaction was conducted according to the general procedure in the presence of benzenediazonium tetrafluoroborate **5c** (0.3 mmol, 58 mg), isoprenyl acetate **6** (4.5 mmol, 450 mg), and photocatalyst **FIC** (0.03 mmol, 11 mg) dissolved in 670 µL dry DMF. The mixture was irradiated for 2 h and the product was obtained after purification using flash chromatography on silica gel, leading to phenylacetone **7c** (condition a: 35 mg, 0.28 mmol, 88% yield) as a white powder.

^{1}H NMR (400 MHz; DMSO-d$_6$) δ: 7.27-7.35 (m, 2H), 7.20-7.27 (m, 1H), 7.13-7.20 (m, 2H), 3.75 (s, 2H), 2.12 (s, 3H).

2-Bromophenylacetone (7d)[7]

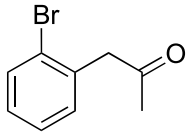

C_9H_9BrO

Mol. Wt: 213.07

7d

The reaction was conducted according to the general procedure in the presence of 2-bromobenzenediazonium tetrafluoroborate **5d** (0.3 mmol, 82 mg), isoprenyl acetate **6** (4.5 mmol, 450 mg), and photocatalyst **FIC** (0.03 mmol, 11 mg) dissolved in 670 µL dry DMF. The mixture was irradiated for 4 h and the product was obtained after purification using flash chromatography on silica gel, leading to 2-bromophenylacetone **7d** (condition a: 39 mg, 0.18 mmol, 61% yield) as a yellow oil.

^{1}H NMR (400 MHz; CDCl$_3$) δ: 7.60 (d, *J* = 8.1, 1H), 7.27-7.39 (m, 2H), 7.20 (td, *J* = 8.0 and 2.3, 1H), 3.93 (s, 2H), 2.18 (s, 3H).

[7] Hisano, N.; Kamei, Y.; Kansaku, Y.; Yamanaka, M.; Mori, K. Construction of 1, 3-Dithio-substituted Tetralins by [1, 5]-alkylthio group transfer mediated skeletal rearrangement. *Org. Lett.* **2018**, *20*, 4223–4226.

2-Cyanophenylacetone (7e)[8]

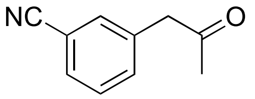

$C_{10}H_9NO$

Mol. Wt: 159.19

7e

The reaction was conducted according to the general procedure in the presence of 2-cyanobenzenediazonium tetrafluoroborate **5e** (0.3 mmol, 65 mg), isoprenyl acetate **6** (4.5 mmol, 450 mg), and photocatalyst **FIC** (0.03 mmol, 11 mg) dissolved in 670 µL dry DMF. The mixture was irradiated for 4 h and the product was obtained after purification using flash chromatography on silica gel, leading to 2-cyanophenylacetone **7e** (condition a: 35 mg, 0.22 mmol, 75% yield) as a brown powder.

^{1}H NMR (400 MHz; $CDCl_3$) δ: 7.69-7.74 (m, 1H), 7.63-7.66 (m, 1H), 7.50-7.54 (m, 2H), 3.90 (s, 2H), 2.18 (s, 3H).

2 Photopolymerization profiles of TA

[8] Wang, L.; Shen, J.; Yang, S.; Liu, W.; Chen, Q.; He, M. C–H arylation reactions through aniline activation catalysed by a PANI-gC 3 N 4-TiO 2 composite under visible light in aqueous medium. *Green Chem.* **2018**, *20*, 1290–1296.

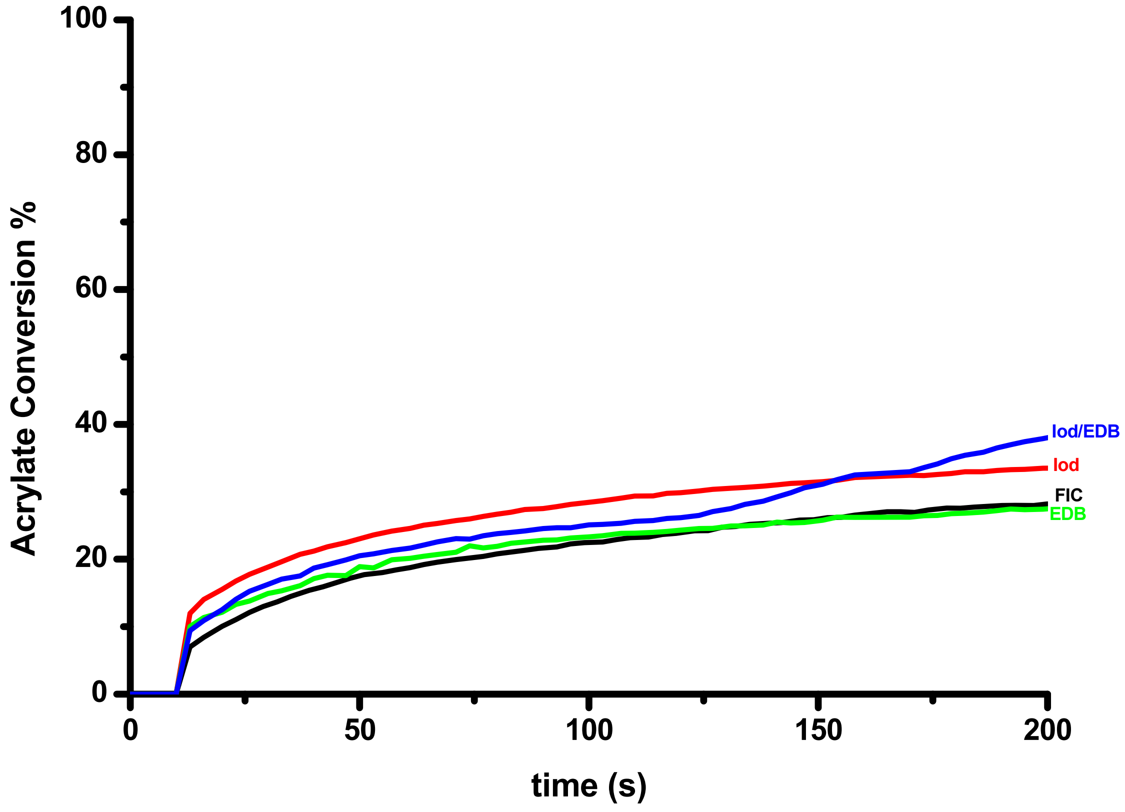

Figure S1. Photopolymerization profiles of TA (acrylate function conversion vs. irradiation time) in laminate (thickness = 25 µm) upon exposure to a LED (λ = 365 nm) in the presence of **FIC** (0.5%w/w), Iod (1%w/w), EDB (1%w/w), and Iod/EDB (1%/1% w/w). The irradiation starts at t = 10 s.

3 Fluorescence quenching study of FIC (by acetophenone, anisole, and *o*-xylene)

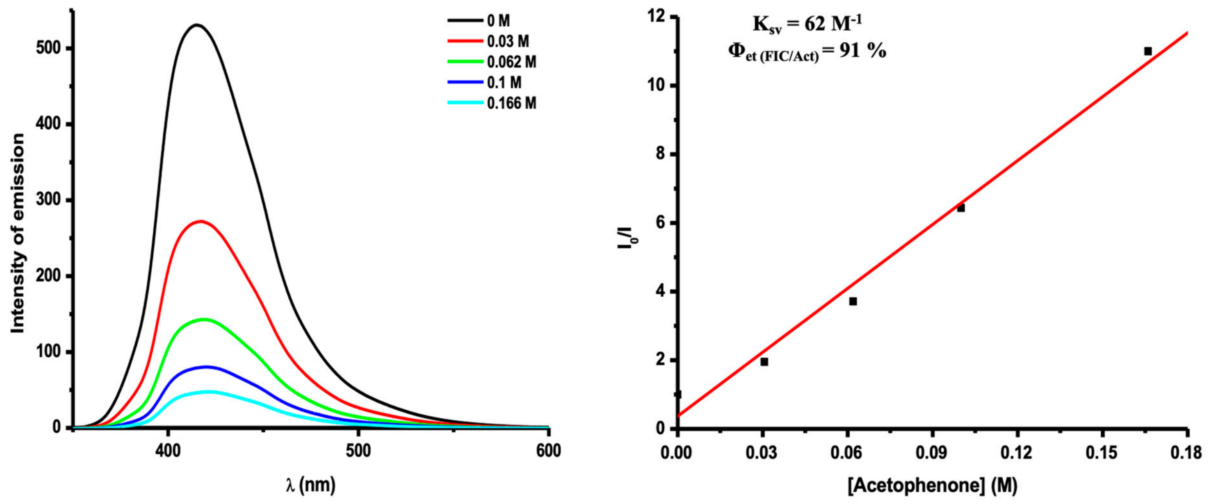

Figure S2. Fluorescence quenching study of **FIC** using acetophenone. Associated Stern–Volmer plot of **FIC** with acetophenone (λ_{exc} = 360 nm).

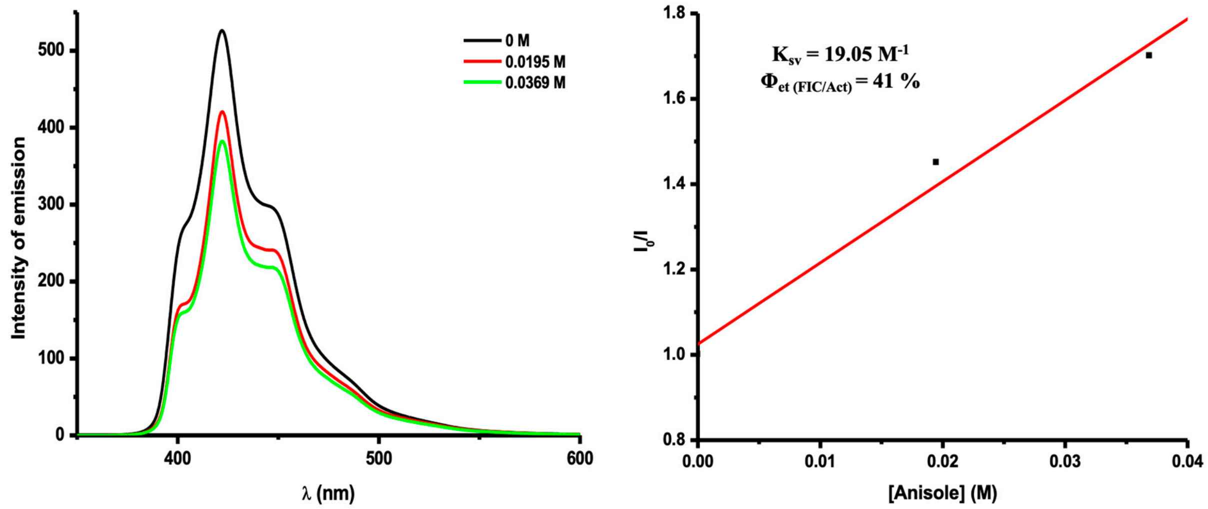

Figure S3. Fluorescence quenching study of **FIC** using anisole in cyclohexane (λ_{exc} = 360 nm). Associated Stern–Volmer plot of **FIC** with anisole.

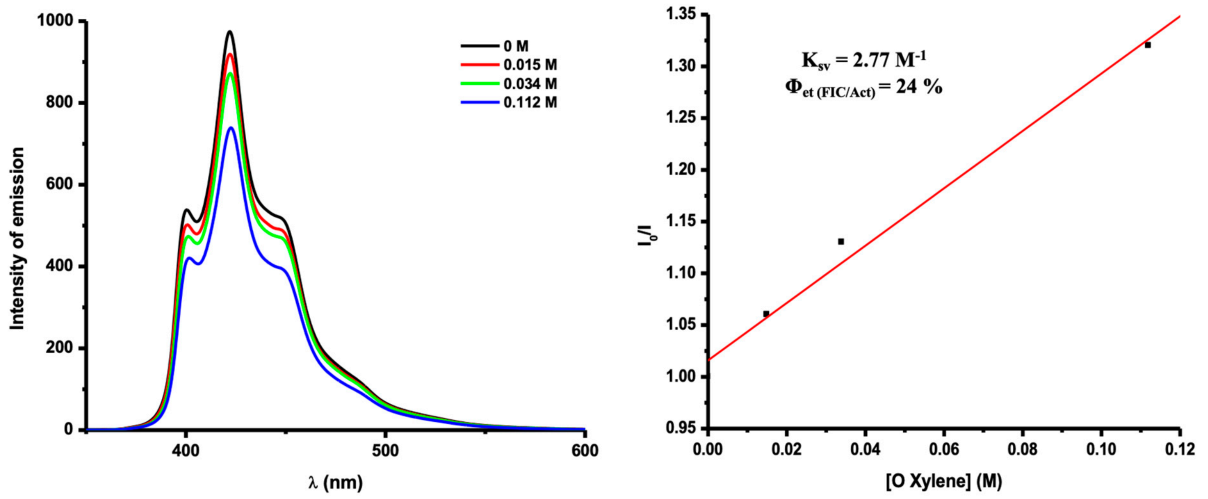

Figure S4. Fluorescence quenching study of **FIC** using *o*-xylene (λ_{exc} = 360 nm). Associated Stern–Volmer plot of **FIC** with *o*-xylene.

PI	E_{S1} (eV)	E_{ox} (V)	ΔG_{S1} (FIC/Acetophenone) (eV)	$K_{sv\ (Act)}$ (M^{-1})	$\Phi_{et\ (FIC/Act)}$	E_{red} (V)	ΔG_{S1} (FIC/Anisole) (eV)	$\Delta G_{S1(FIC/O\text{-}xylene)}$ (eV)	$K_{sv\ (Anisole)}$ (M^{-1})	Φ_{et} (FIC/Anisole)	$K_{sv\ (O\text{-}xylene)}$ (M^{-1})	$\Phi_{et\ (FIC/O\text{-}xylene)}$
FIC	3.27	0.86	- 0.3	62	0.91	-0.63	-0.89	- 0.42	19	0.41	2.77	0.24

Table S1. Parameters characterizing **FIC**/acetophenone interaction (Figure S2) in acetonitrile and **FIC**/anisole (Figure S3) and **FIC**/*o*-xylene (Figure S4) interactions in cyclohexane. Evaluated from:

$\Delta Get = Eox - Ered - E^* + C$

$\Delta G_{et} = E_{ox(FIC)} - E_{red(Acetophenone)} - E^* = 0.86 + 2.11 - 3.27 = - 0.3$ eV

$\Delta G_{et} = E_{ox(Anisole)} - E_{red(FIC)} - E^* = 1.75 + 0.63 - 3.27 = - 0.89$ eV

$\Delta G_{et} = E_{ox(o\text{-Xylene})} - E_{red(FIC)} - E^* = 2.22 + 0.63 - 3.27 = - 0.42$ eV

$E_{red(Acetophenone)} = -2.11$ V

$E_{ox(Anisole)} = +1.75$ V

$E_{ox(o\text{-Xylene})} = +2.22$ V

4 ^{1}H and ^{13}C{^{1}H} NMR Spectra for 3, FIC, and 5e. ^{1}H for Spectra 5a-d, 7a-e.

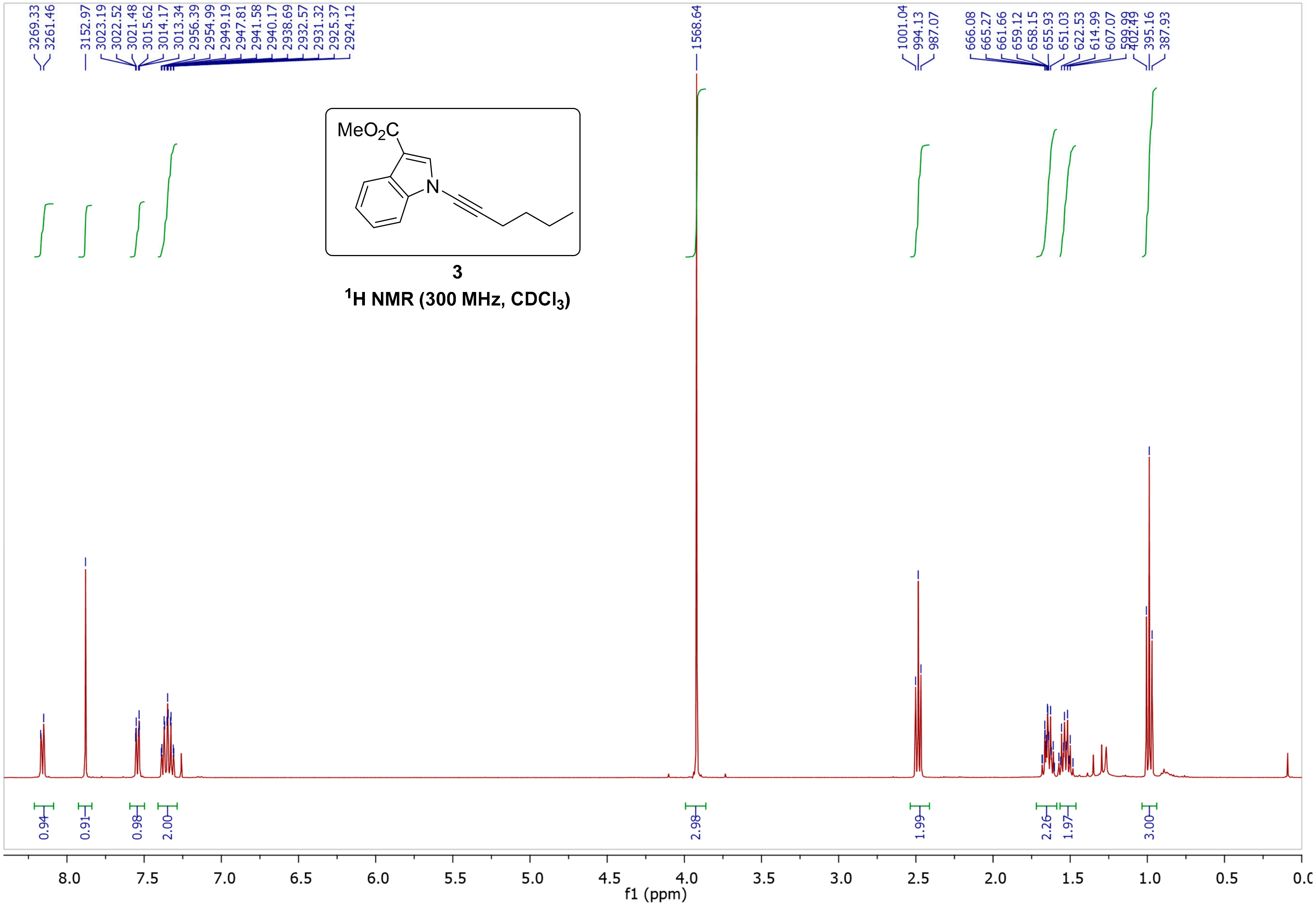
MeO2C
N
3
1H NMR (300 MHz, CDCl3)
3269.33
3261.46
3152.97
3023.19
3022.52
3021.48
3015.62
3014.17
3013.34
2956.39
2954.99
2949.19
2947.81
2941.58
2940.17
2938.69
2932.57
2931.32
2925.37
2924.12
1568.64
1001.04
994.13
987.07
666.08
665.27
661.66
659.12
658.15
655.93
651.03
622.53
614.99
607.07
395.16
387.93
0.94
0.91
0.98
2.00
2.98
1.99
2.26
1.97
3.00
8.0
7.5
7.0
6.5
6.0
5.5
5.0
4.5
4.0
3.5
3.0
2.5
2.0
1.5
1.0
0.5
f1 (ppm)

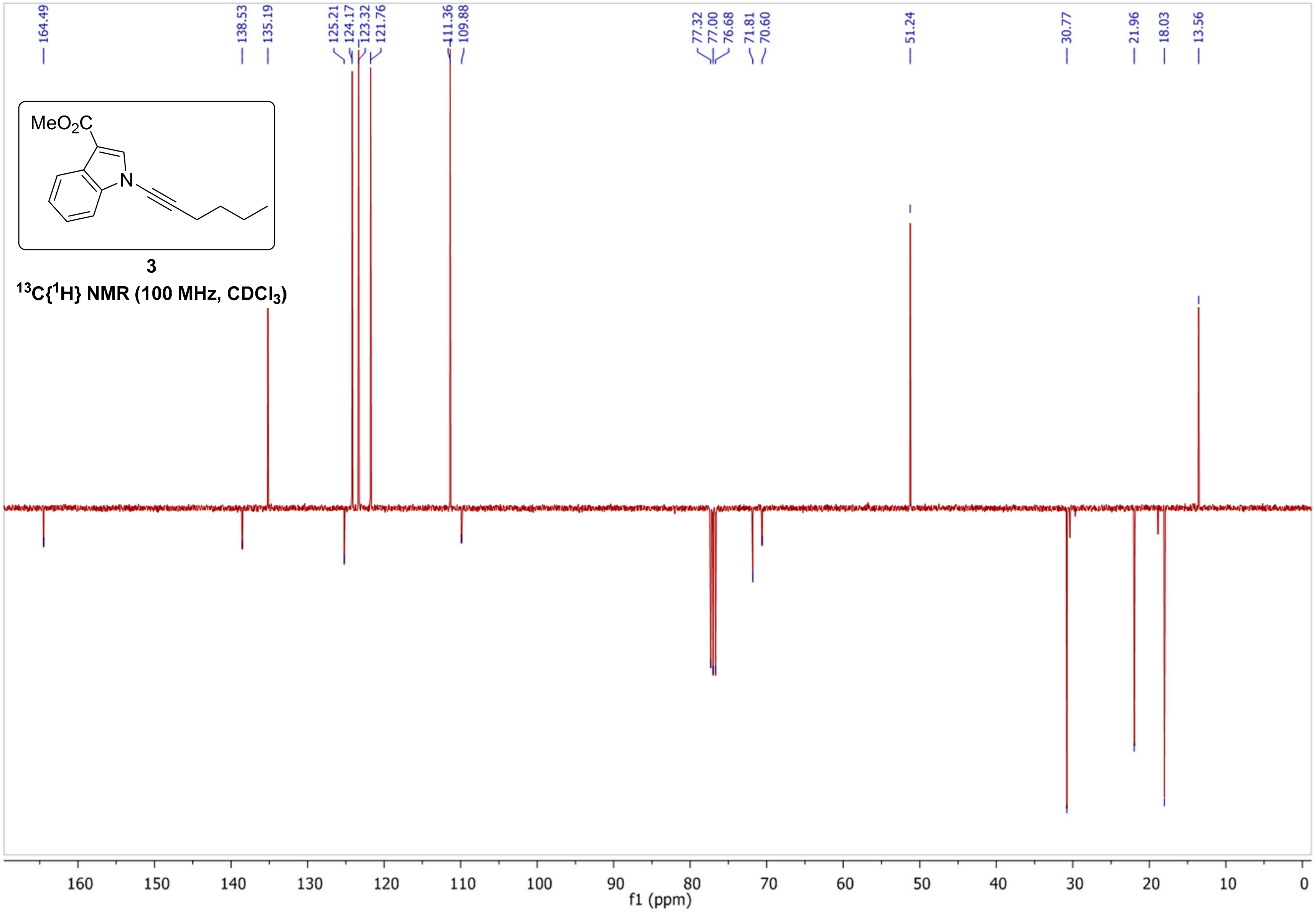

3

$^{13}C\{^{1}H\}$ NMR (100 MHz, $CDCl_3$)

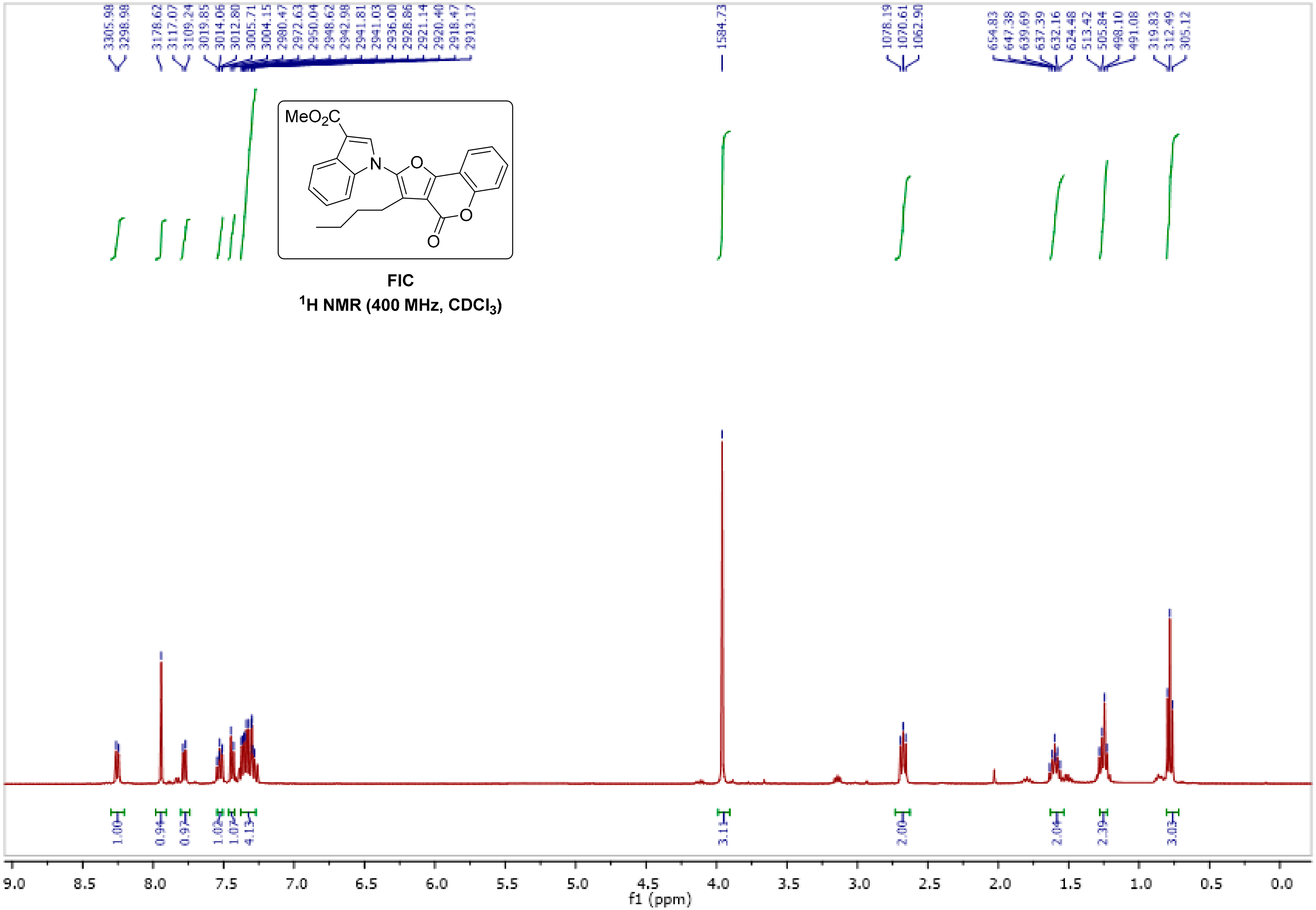
MeO2C
N
O
O
O
FIC
1H NMR (400 MHz, CDCl3)
f1 (ppm)

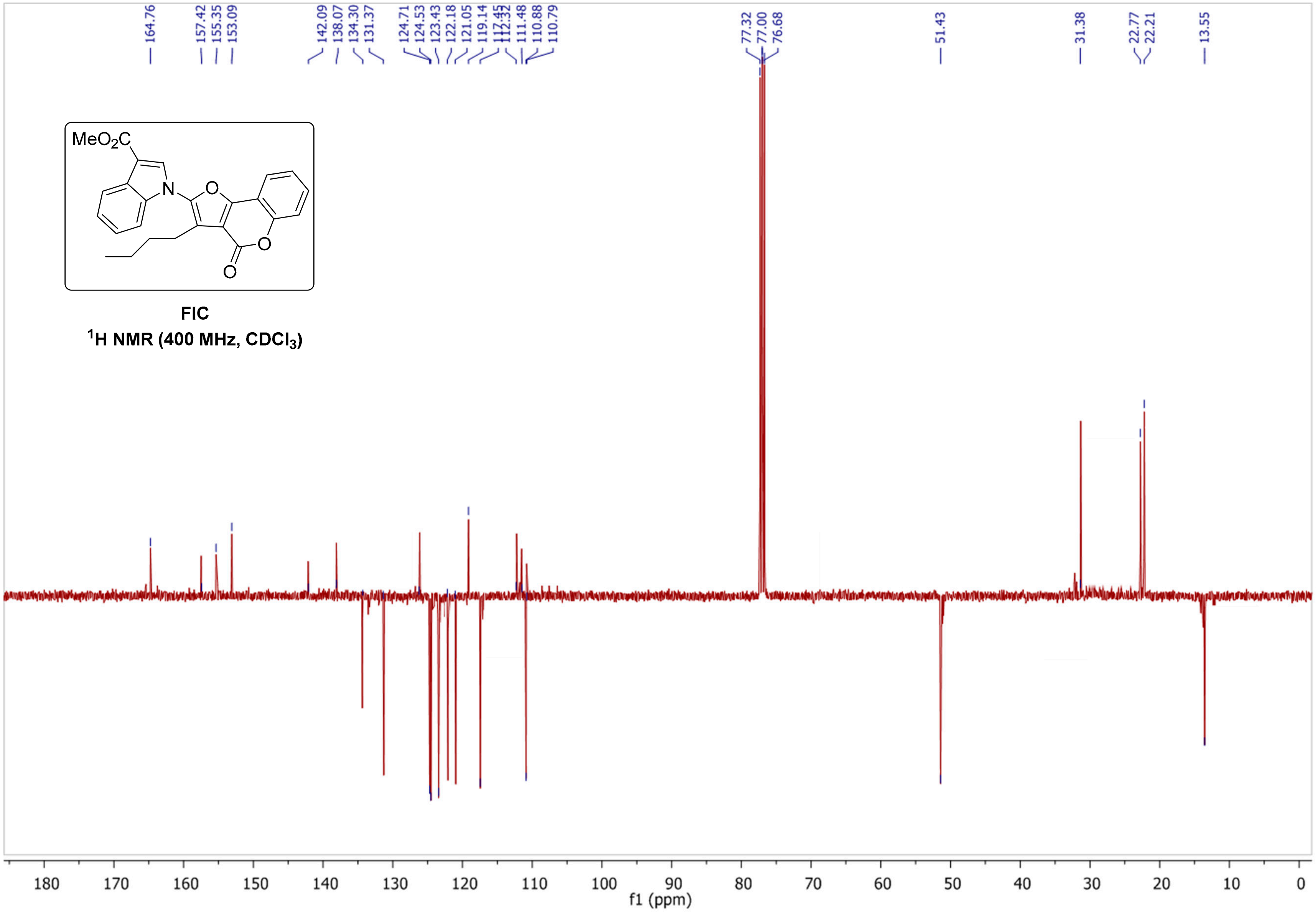
164.76
157.42
155.35
153.09
142.09
138.07
134.30
131.37
124.71
124.53
123.43
122.18
121.05
119.14
111.48
110.88
110.79
77.32
77.00
76.68
51.43
31.38
22.77
22.21
13.55
MeO2C
N
O
O
O
FIC
1H NMR (400 MHz, CDCl3)
180
170
160
150
140
130
120
110
100
90
80
70
60
50
40
30
20
10
0
f1 (ppm)

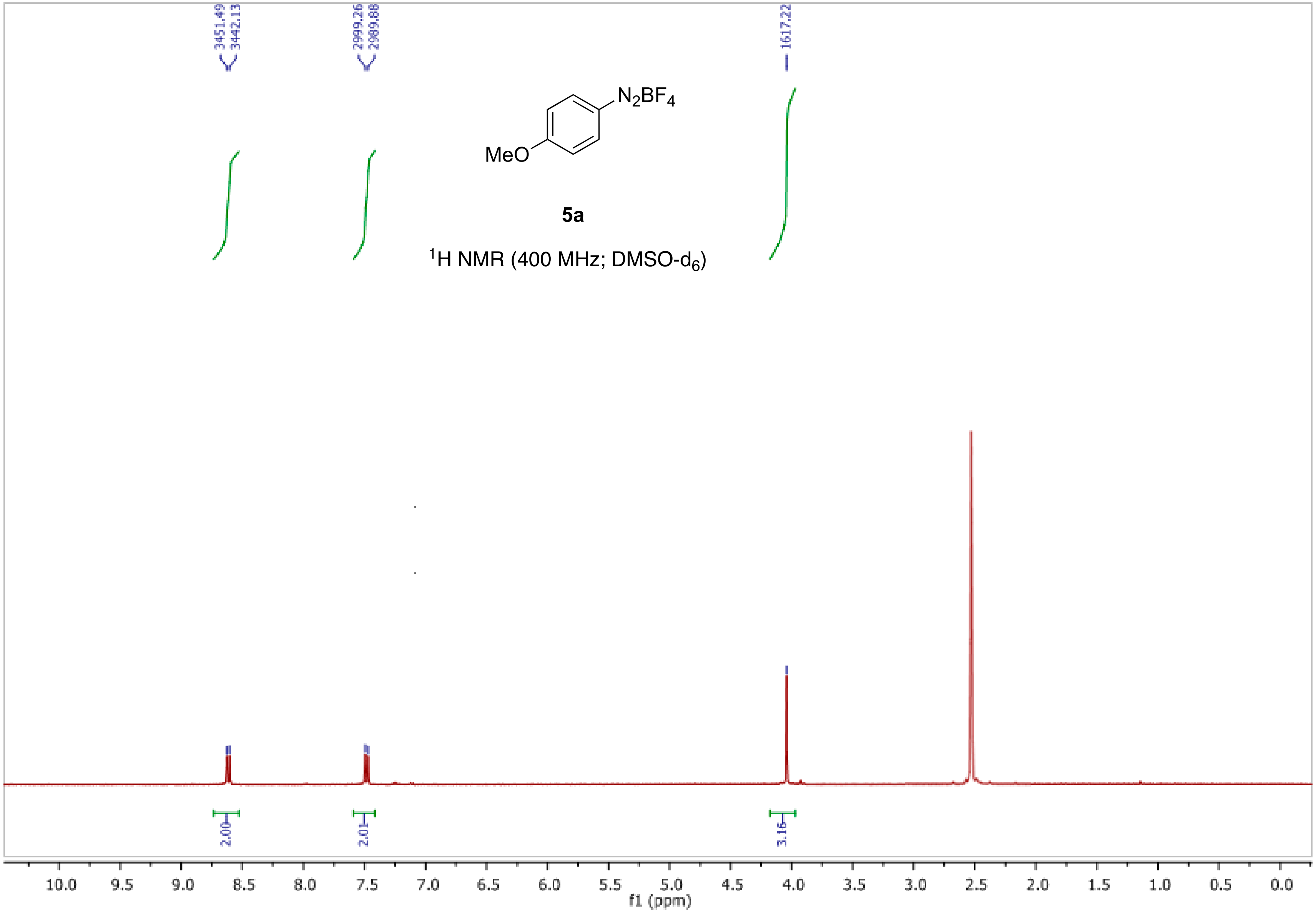

3451.49
3442.13
2999.26
2989.88
1617.22
N2BF4
MeO
5a
1H NMR (400 MHz; DMSO-d6)
2.00
2.01
3.16
10.0
9.5
9.0
8.5
8.0
7.5
7.0
6.5
6.0
5.5
5.0
4.5
4.0
3.5
3.0
2.5
2.0
1.5
1.0
0.5
0.0
f1 (ppm)

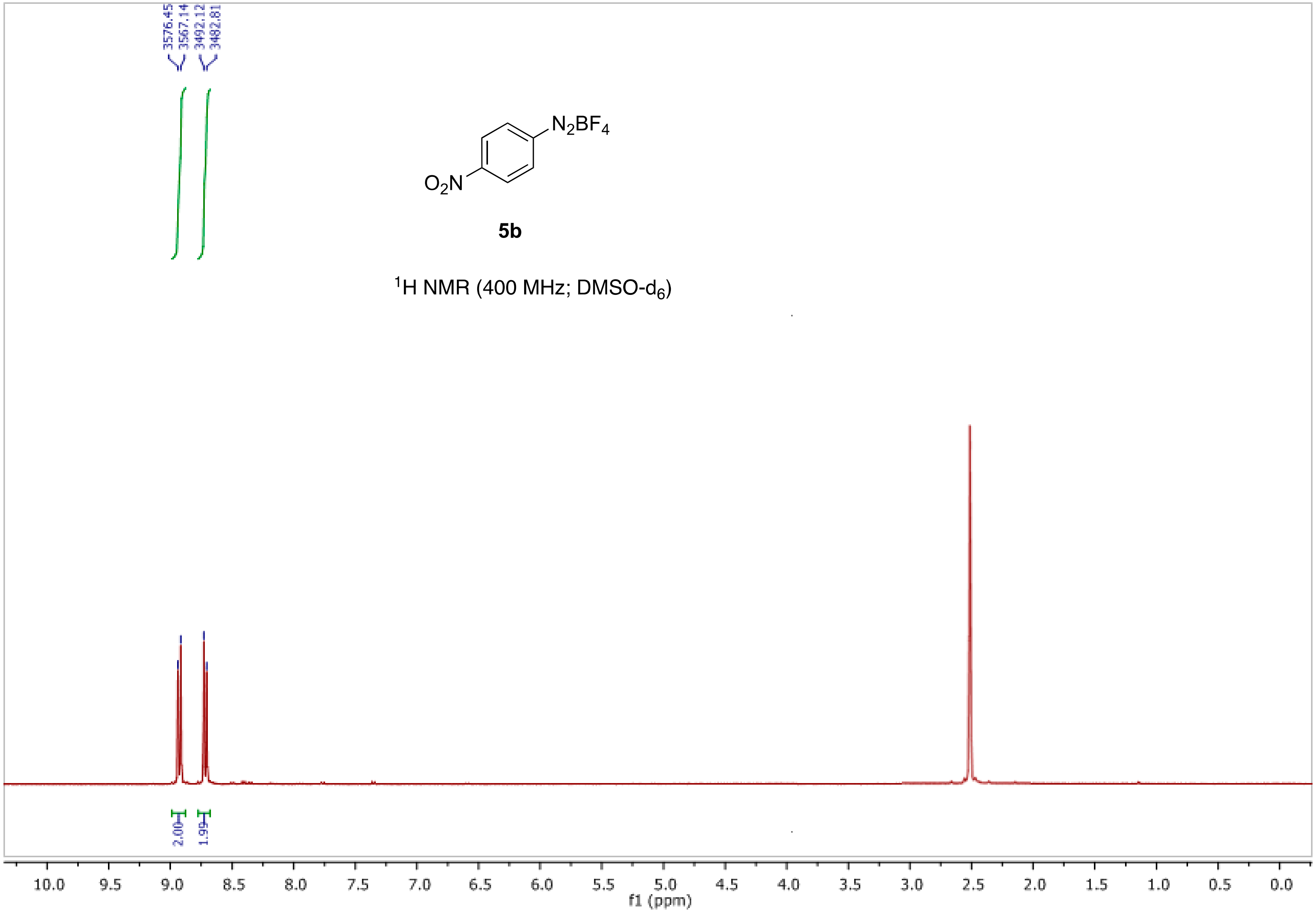

3576.45
3567.14
3492.12
3482.81
N2BF4
O2N
5b
1H NMR (400 MHz; DMSO-d6)
2.00
1.99
10.0
9.5
9.0
8.5
8.0
7.5
7.0
6.5
6.0
5.5
5.0
4.5
4.0
3.5
3.0
2.5
2.0
1.5
1.0
0.5
0.0
f1 (ppm)

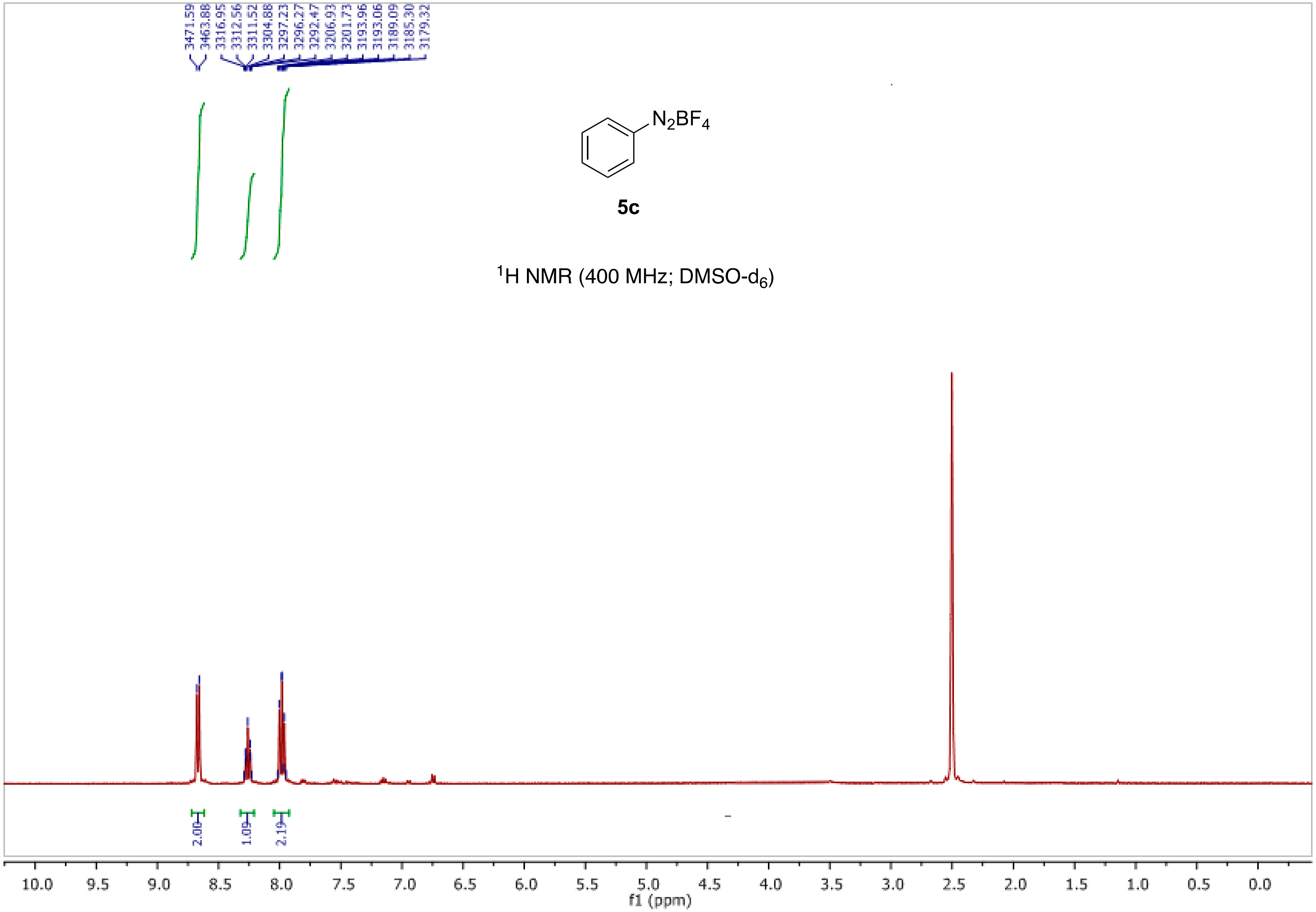
3471.59
3463.88
3316.95
3312.56
3311.52
3304.88
3297.23
3296.27
3292.47
3206.93
3201.73
3193.96
3193.06
3189.09
3185.30
3179.32
N2BF4
5c
1H NMR (400 MHz; DMSO-d6)
2.00
1.09
2.19
10.0
9.5
9.0
8.5
8.0
7.5
7.0
6.5
6.0
5.5
5.0
4.5
4.0
3.5
3.0
2.5
2.0
1.5
1.0
0.5
0.0
f1 (ppm)

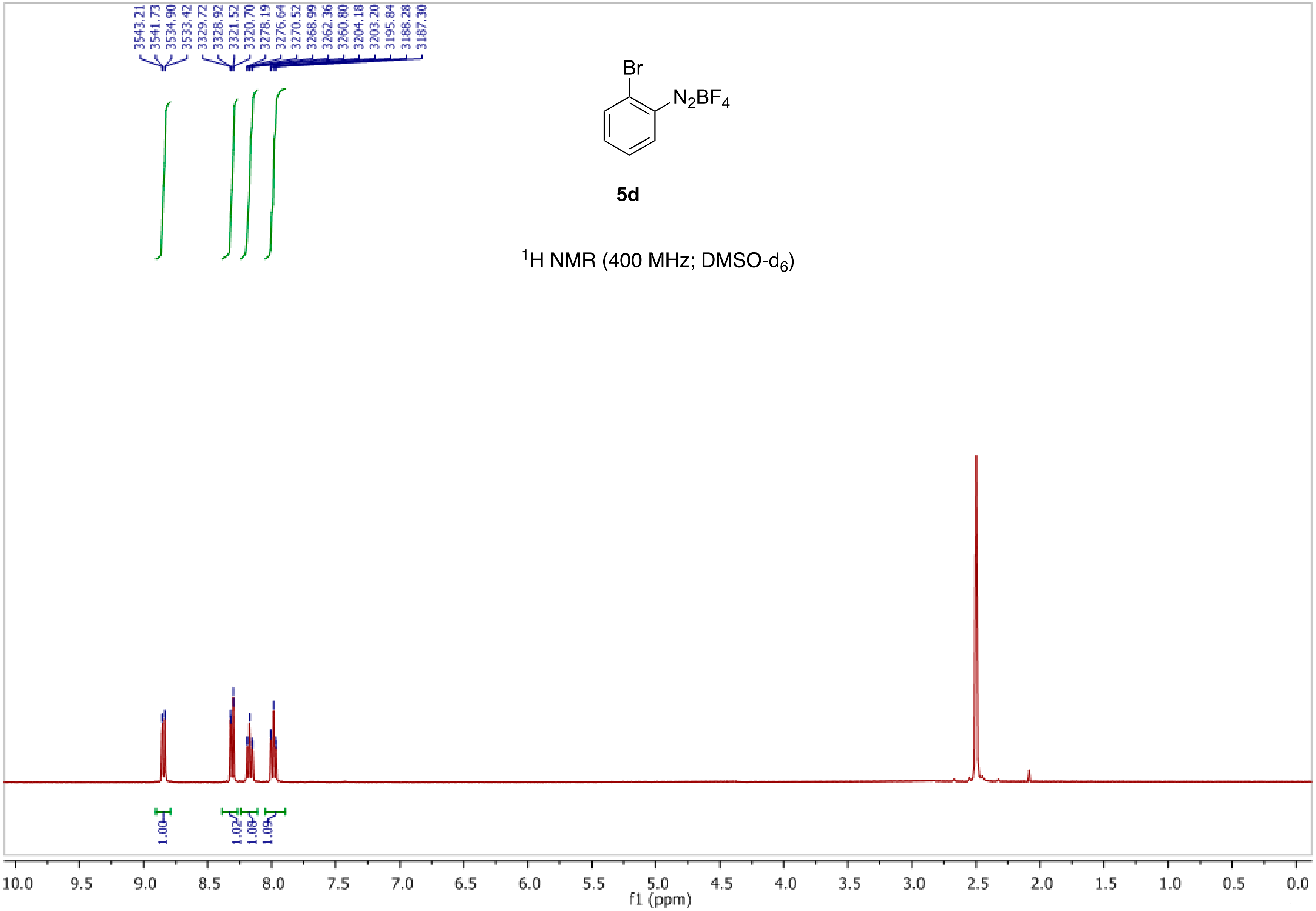
3543.21
3541.73
3534.90
3533.42
3329.72
3328.92
3321.52
3320.70
3278.19
3276.64
3270.52
3268.99
3262.36
3260.80
3204.18
3203.20
3195.84
3188.28
3187.30
Br
N2BF4
5d
1H NMR (400 MHz; DMSO-d6)
1.00
1.02
1.08
1.09
10.0
9.5
9.0
8.5
8.0
7.5
7.0
6.5
6.0
5.5
5.0
4.5
4.0
3.5
3.0
2.5
2.0
1.5
1.0
0.5
0.0
f1 (ppm)

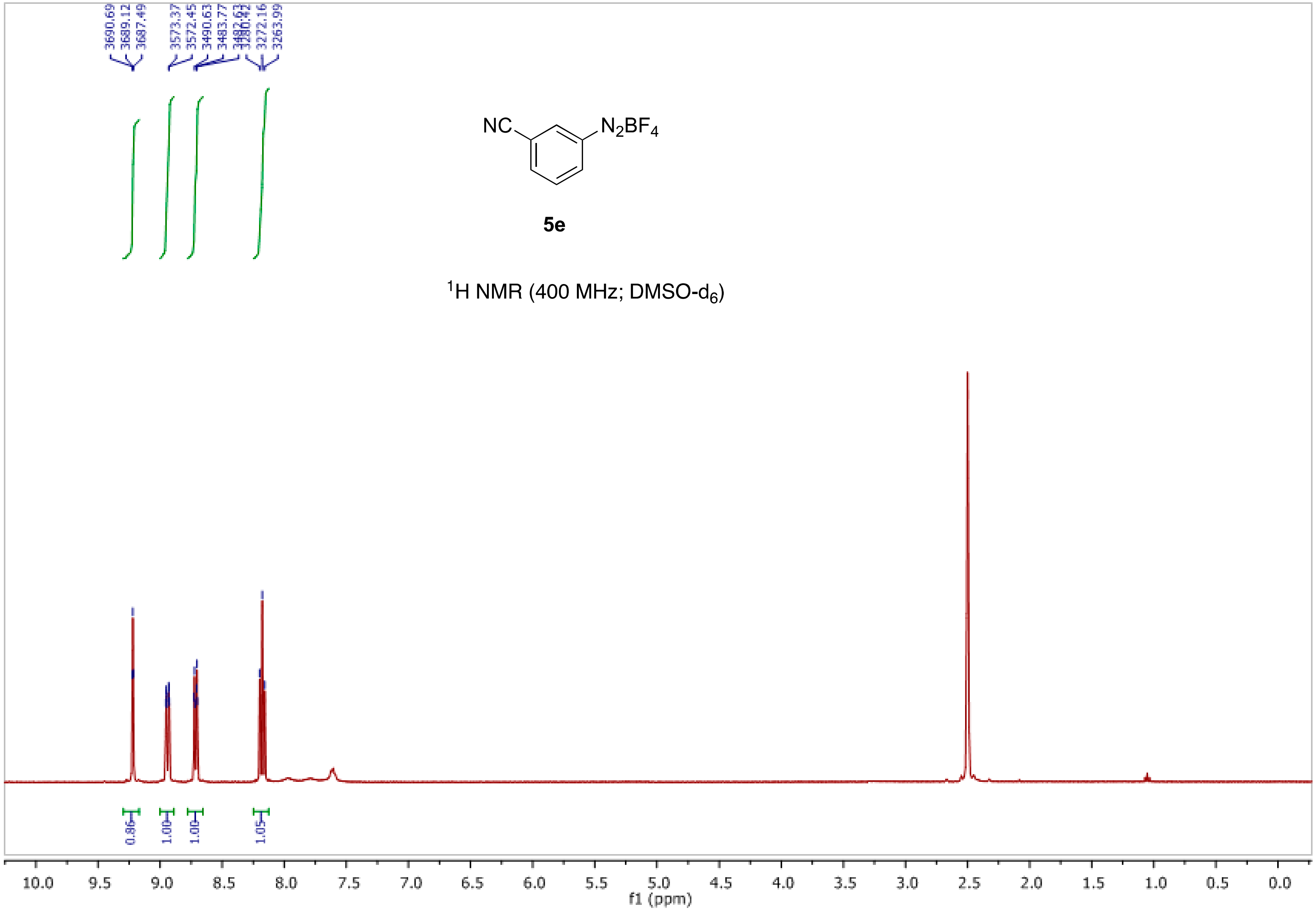

NC
N2BF4
5e
1H NMR (400 MHz; DMSO-d6)
f1 (ppm)

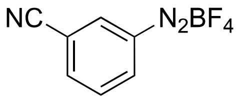

5e

$^{13}C\{^{1}H\}$ NMR (100 MHz; DMSO-d$_6$)

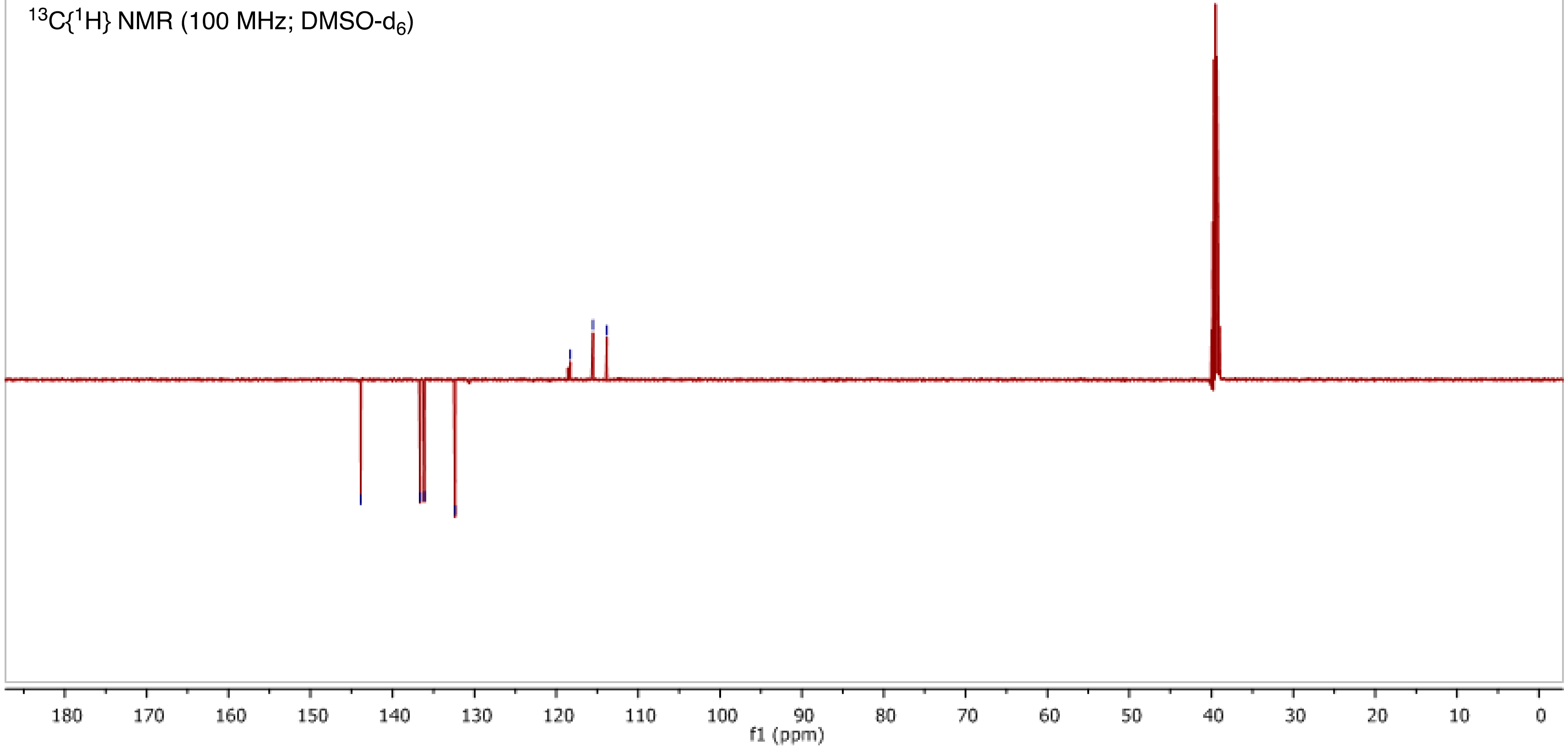

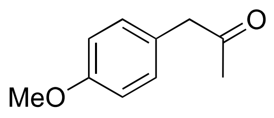

7a

^{1}H NMR (400 MHz; DMSO-d$_6$)

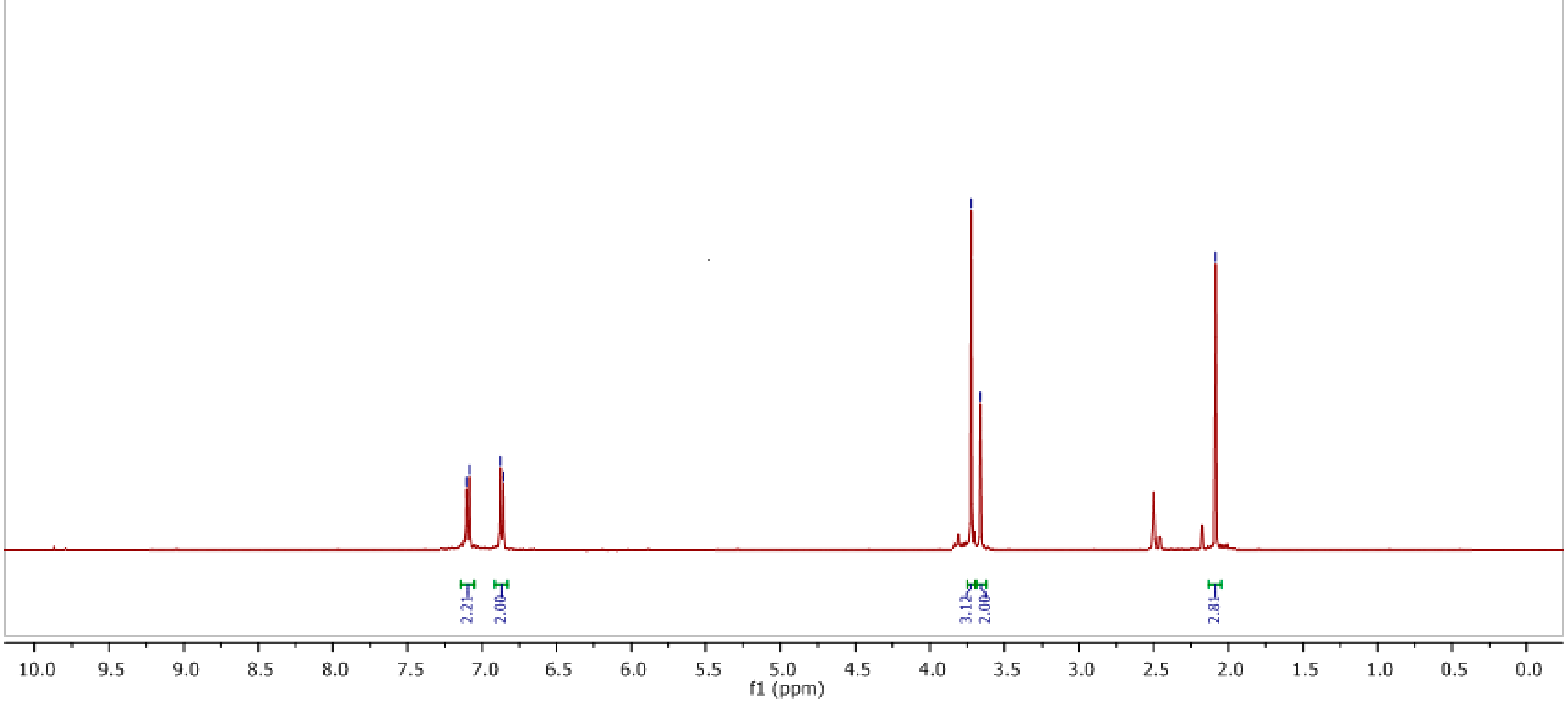

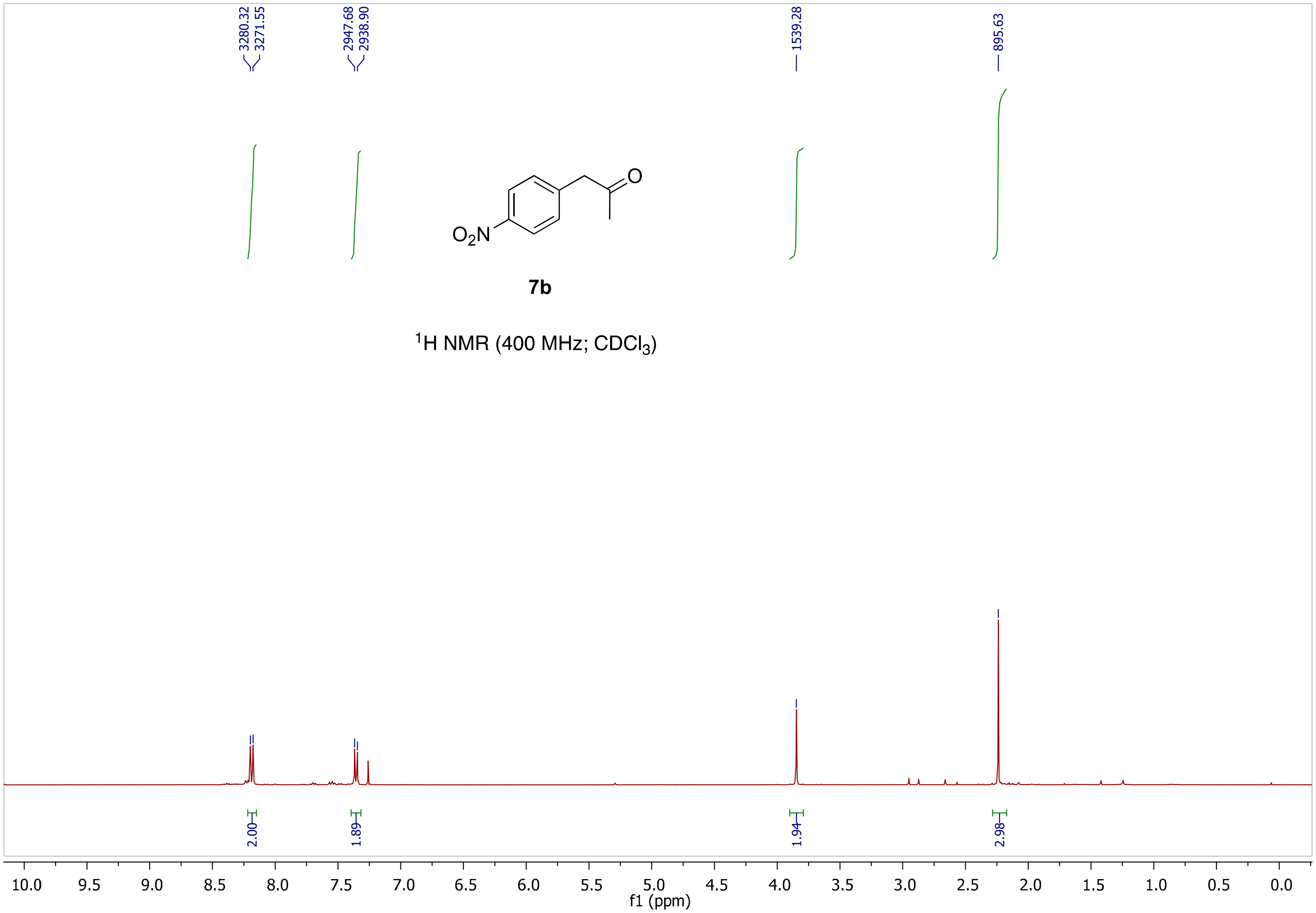
3280.32
3271.55
2947.68
2938.90
1539.28
895.63
O2N
O
7b
1H NMR (400 MHz; CDCl3)
2.00
1.89
1.94
2.98
10.0 9.5 9.0 8.5 8.0 7.5 7.0 6.5 6.0 5.5 5.0 4.5 4.0 3.5 3.0 2.5 2.0 1.5 1.0 0.5 0.0
f1 (ppm)

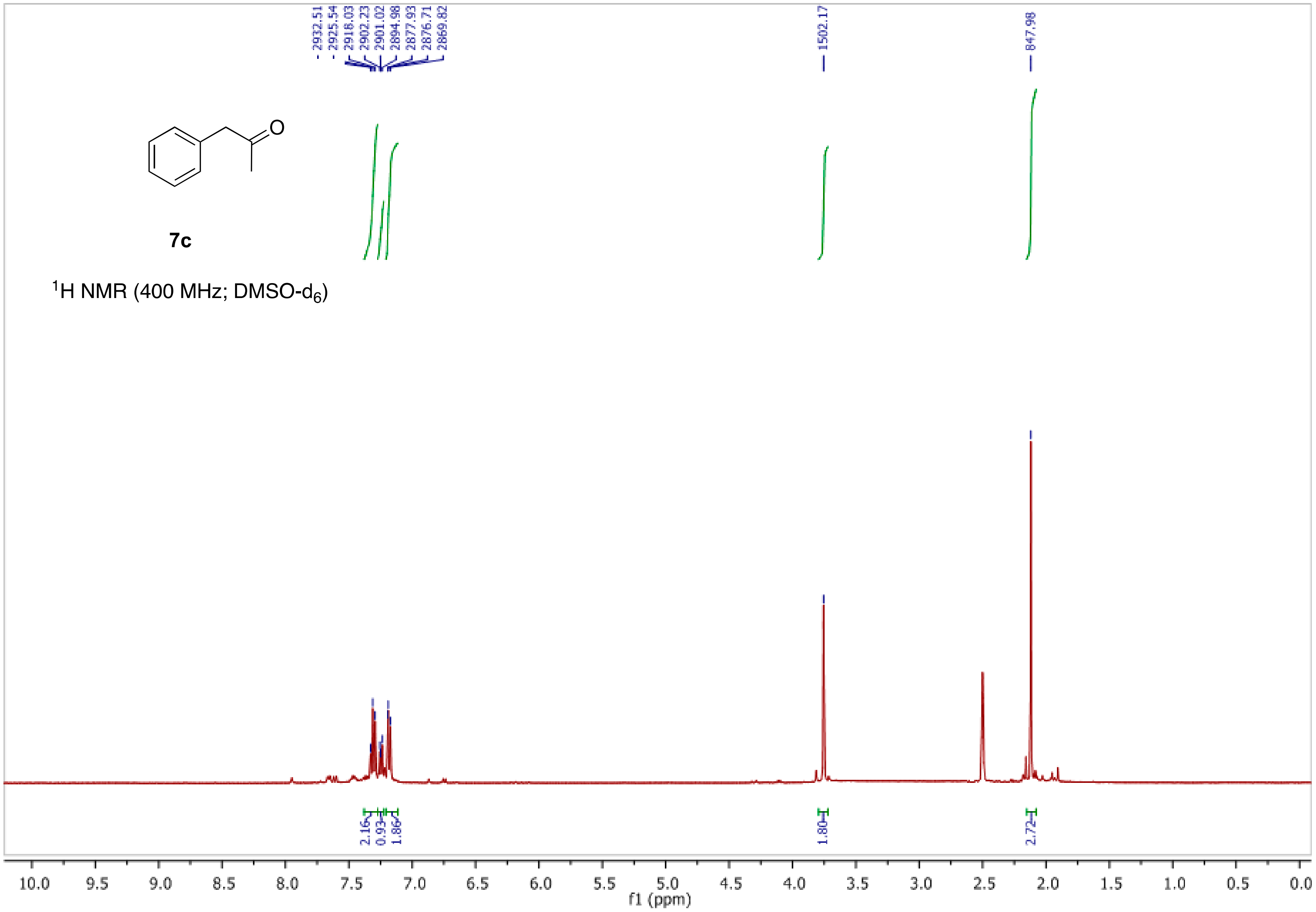
7c
^{1}H NMR (400 MHz; DMSO-d$_6$)
2932.51
2925.54
2918.03
2902.23
2901.02
2894.98
2877.93
2876.71
2869.82
1502.17
847.98
2.16
0.93
1.86
1.80
2.72
10.0 9.5 9.0 8.5 8.0 7.5 7.0 6.5 6.0 5.5 5.0 4.5 4.0 3.5 3.0 2.5 2.0 1.5 1.0 0.5 0.0
f1 (ppm)

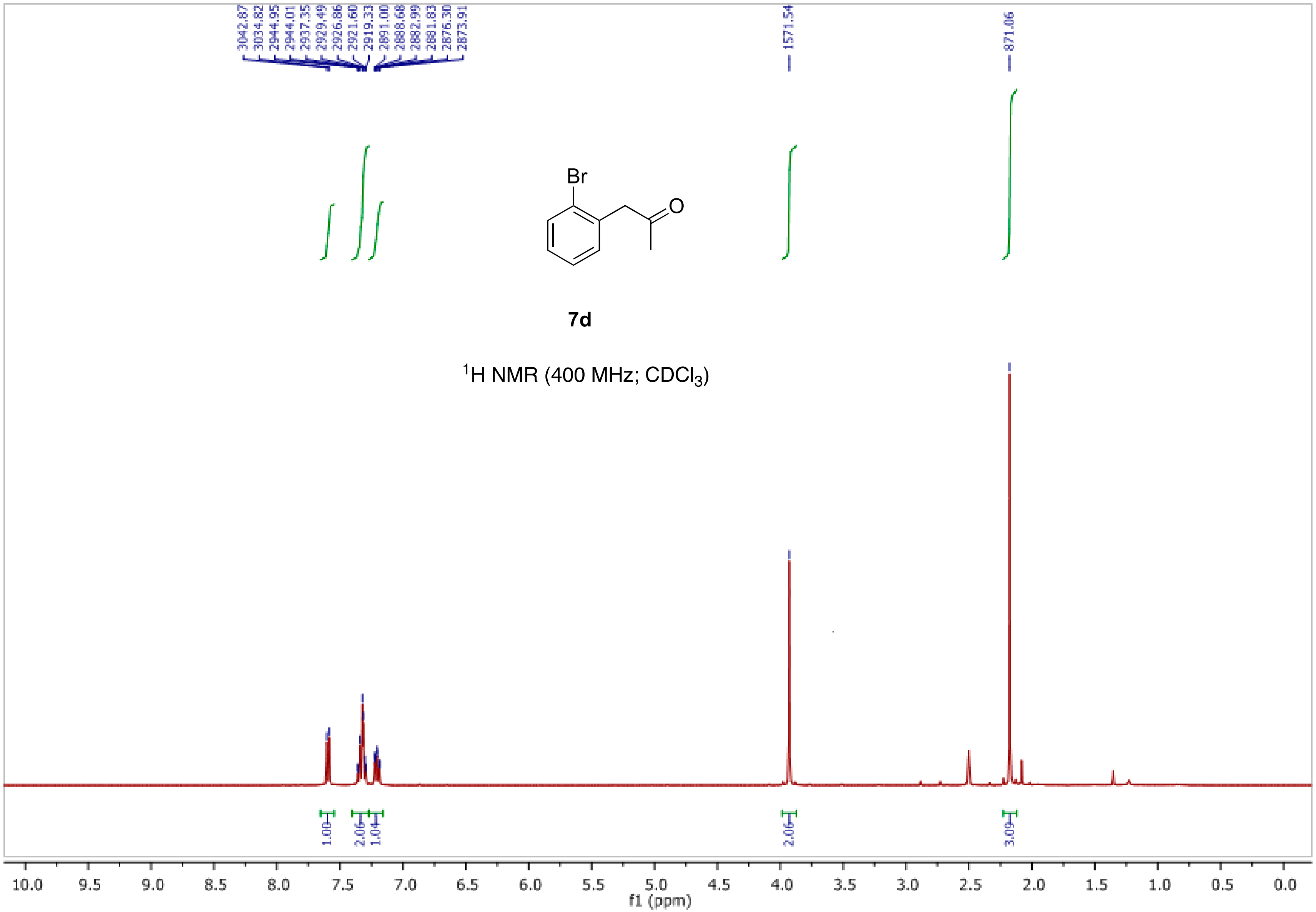
3042.87
3034.82
2944.95
2944.01
2937.35
2929.49
2926.86
2921.60
2919.33
2891.00
2888.68
2882.99
2881.83
2876.30
2873.91
1571.54
871.06
Br
O
7d
^{1}H NMR (400 MHz; $CDCl_3$)
1.00
2.06
1.04
2.06
3.09
10.0 9.5 9.0 8.5 8.0 7.5 7.0 6.5 6.0 5.5 5.0 4.5 4.0 3.5 3.0 2.5 2.0 1.5 1.0 0.5 0.0
f1 (ppm)

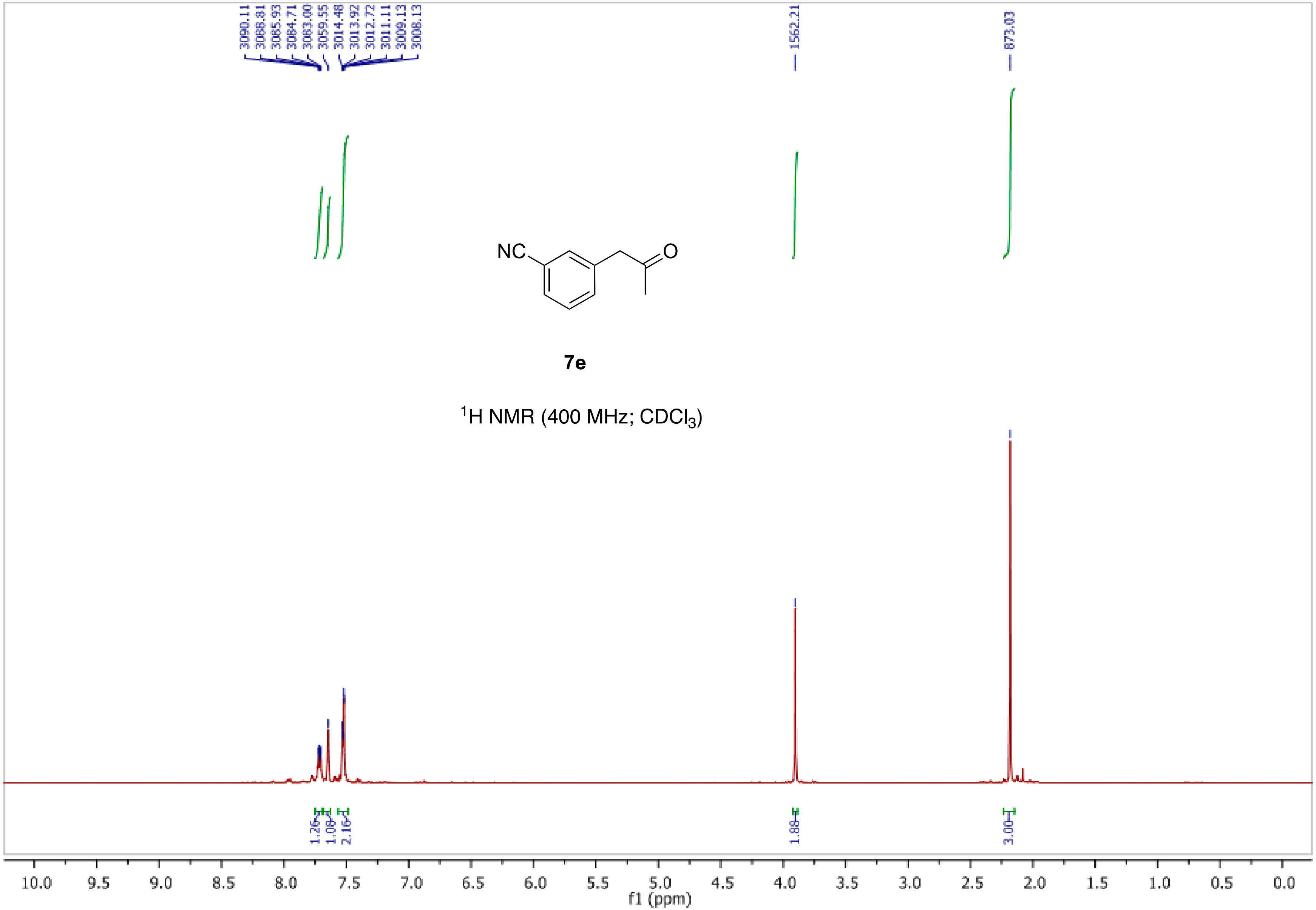
3090.11
3088.81
3085.93
3084.71
3083.00
3059.55
3014.48
3013.92
3012.72
3011.11
3009.13
3008.13
1562.21
873.03
NC
O
7e
^{1}H NMR (400 MHz; $CDCl_3$)
1.26
1.08
2.16
1.88
3.00
10.0 9.5 9.0 8.5 8.0 7.5 7.0 6.5 6.0 5.5 5.0 4.5 4.0 3.5 3.0 2.5 2.0 1.5 1.0 0.5 0.0
f1 (ppm)
